# Ageing-driven molecular and functional changes in the bovine endometrium

**DOI:** 10.1371/journal.pone.0332176

**Published:** 2025-09-26

**Authors:** Marine Denis, Doulaye Dembélé, Christophe Richard, Marie Leduc, Maud Pez, Pierrette Reinaud, Olivier Dubois, Hélène Kiefer, Hélène Jammes, Valérie Gelin, Fabienne Constant, François Vialard, Gilles Foucras, Pierre Germon, Gilles Charpigny, Olivier Sandra, Mariam Raliou

**Affiliations:** 1 Université Paris-Saclay, UVSQ, INRAE, BREED, Jouy-en-Josas, France; 2 Ecole Nationale Vétérinaire d’Alfort, BREED, Maisons-Alfort, France; 3 Institut de Génétique et de Biologie Moléculaire et Cellulaire, CNRS UMR – Inserm U 964 – Université de Strasbourg, Illkirch, France; 4 UFR-SVS, UVSQ, Montigny le Bretonneux, France; 5 IHAP, Université de Toulouse, INRAE, ENVT, Toulouse, France; 6 ISP UMR, INRAE, Université François Rabelais de Tours, Nouzilly, France; University of Hawai'i at Manoa, UNITED STATES OF AMERICA

## Abstract

Understanding how ageing impacts endometrial function is crucial for preserving fertility in older females. While ageing-related cellular dysfunction and inflammation are observed in the bovine uterus, its effects on endometrial physiology remain unclear. Using an experimental model of young (4−7 years) and old (13−15 years) cloned female cattle, we assessed the effect of ageing on the endometrium through transcriptome profiling and responses of cultured endometrial cells to interferon tau (IFNT) and of cultured endometrial explants to LPS. Progesterone profiles were similar between young and old females. Transcriptomic analysis of endometrial biopsies on day 15 of the estrous cycle identified 859 differentially expressed genes (DEG; p ≤ 0.05, |FC| ≥ 1.5), among which 402 DEG were over-expressed and 457 DEG were under-expresssed in old females. These DEG are linked to immune, inflammatory, metabolic, and cell organization pathways and networks. RT-qPCR validation of selected candidate DEG revealed an increased expression of *COL4A3*, *CPA3*, *IGFBP1*, *IGFBP2*, *RSAD2*, *SCARA5*, and *SERPINA14* in young females. *In vitro* stimulation with IFNT of primary uterine glandular epithelial and stromal cells revealed that glandular epithelial cells exhibit a greater sensitivity to IFNT than stromal cells, both in old and young females. Glandular epithelial cells derived from old females exhibit a weaker response to IFNT, in terms of the number of differentially expressed genes, compared to those from young females. The effect of LPS treatment on cytokine concentrations was lightly more pronounced in young females than in old females, with LPS leading to a significant increase in the concentration of IL-1α, IL-1β, IL-6 and IL-10 in young females. By altering the transcriptomic profile of the endometrium and its capacity to respond to both the embryo’s signal and inflammatory factors, we propose that age may be a key factor underlying uterine-related reproductive failures. Further experiments are required to confirm this hypothesis.

## Introduction

The uterus is essential and irreplaceable for the establishment and maintenance of a successful pregnancy that ends with the birth of living and healthy offspring. Pregnancy results from intricate, reciprocal interactions between the embryo and the endometrium, starting as early as the late pre-implantation phase. While optimal embryo quality is a prerequisite, most embryo losses observed during pregnancy are linked to inadequate interactions between the implanting conceptus and the endometrium [1–3]. The dynamic property of the endometrium as a sensor of embryo quality has been first demonstrated in cattle and later confirmed in humans [4,5]. Alterations of the sensor-driver properties of the endometrium significantly affect implantation, placentation and foetal development, with post-natal repercussions [6–8].

Endometrial physiology is influenced by intrinsic factors such as metabolism, inflammation, and psychological stress as well as extrinsic factors, including biotic and abiotic factors such as heat, nutrition, exogenous hormones, and infections [8–13]. Among the intrinsic factors, ageing—a process often accompanied by chronic inflammation—is associated with a decline in organ function [14].

Ageing is notably characterized by an accumulation of senescent cells, a weakened immune response, metabolic dysfunction and tissue fibrosis [15–18]. Ageing is also associated with a decline in female reproduction capacity. In rodents, maternal ageing is linked to increased litter heterogeneity and a rise in developmental anomalies [7]. In alpacas and cows, embryo transfer success rates decrease as the recipient’s age increases [19,20]. While age-related ovulatory dysfunction and reduced oocyte quality have been well documented across many species [21–23], the impact of ageing on the structural organization and gene expression profiles of the endometrium has received comparatively less attention. For example, women over 45 exhibit more pronounced signs of cellular senescence in the uterus compared with younger women [24]. In cows, the incidence and severity of adenomyosis increase with age [25]. However, the effects of ageing on endometrial function, particularly the ability of this tissue to interact with the embryo during implantation or to cope with inflammation, remain largely unexplored.

Understanding the molecular and biological mechanisms underlying uterine ageing appears critical in the context of reproductive health, such as the rising maternal age at first pregnancy in humans and the question of extending longevity to improve productivity of dairy cows in conventional and organics farming systems [26].

Investigating uterine ageing is inherently complex due to the organ’s function and sensitivity to hormonal influences. Factors such as genetic, environmental conditions, parity, and systemic ageing of the maternal organism—including the hypothalamic-pituitary-gonadal axis—introduce significant confounding variables. A research animal model that minimizes genetic diversity and environmental variability represents a relevant option to reduce study biases.

Our study investigates the molecular and biological changes associated with uterine ageing in cattle, using an experimental model comprising bovine females of two distinct ages, all sharing the same nuclear genome and raised under identical farming conditions. Transcriptomic analyses were performed on endometrial biopsies collected on Day 15 (D15) of the estrous cycle during the peri-implantation period when endometrium is receptive to conceptus signals. To assess the functional consequences of endometrial ageing, two *in vitro* experiments were carried out and involved either primary cell cultures or endometrial explant cultures. First, we evaluated the molecular response of primary bovine glandular epithelial and stromal cells to Interferon-tau (IFNT), the key signal of pregnancy recognition in ruminants, at D15. Second, we examined the inflammatory response of endometrial explants exposed to lipopolysaccharide (LPS), a component of the cell wall of Gram-negative bacteria, on Day 3 (D3) of the estrous cycle, a time point chosen to minimize the anti-inflammatory effect of progesterone, which is low at this stage of the cycle.

## Materials and methods

### Animals and sample collection

Experiments were conducted in compliance with European Community Directive 2010/63/EU, which revises Directive 86/609/EEC on the protection of animals used for scientific purposes. The animal procedures associated with endometrial biopsy collection were approved by the Ethical Committee of Animal Experimentation of INRAE and AgroParisTech (CEEA45 “COMETHEA”; reference APAFIS#596) with authorization subsequently granted by the French Ministry of Education and Scientific Research (reference 2015050417061568).

Fifteen Holstein females, derived from somatic nucleus cloning of a single cell donor cow, were included in the study. Females from this cell line were previously used and described in studies investigating the effects of ageing on the inflammatory response in cattle [27]. The use of this somatic clone model minimizes inter-individual genetic variability. Moreover, all females were raised under standardized feeding and housing conditions (https://doi.org/10.17180/MAQZ-V844), limiting environmental effects.

The animals were divided into two groups: young (4–7 years) primiparous females (YF) and old (13–15 years) nulliparous females (OF). Detailed information on the animals is provided in S1 Table. For all females, estrous cycle were synchronised using the Crestar method, as described by [27], with the day of estrus designated as Day 0. For the YF group, endometrial biopsies were collected more than a year after calving.

**Fig 1:**
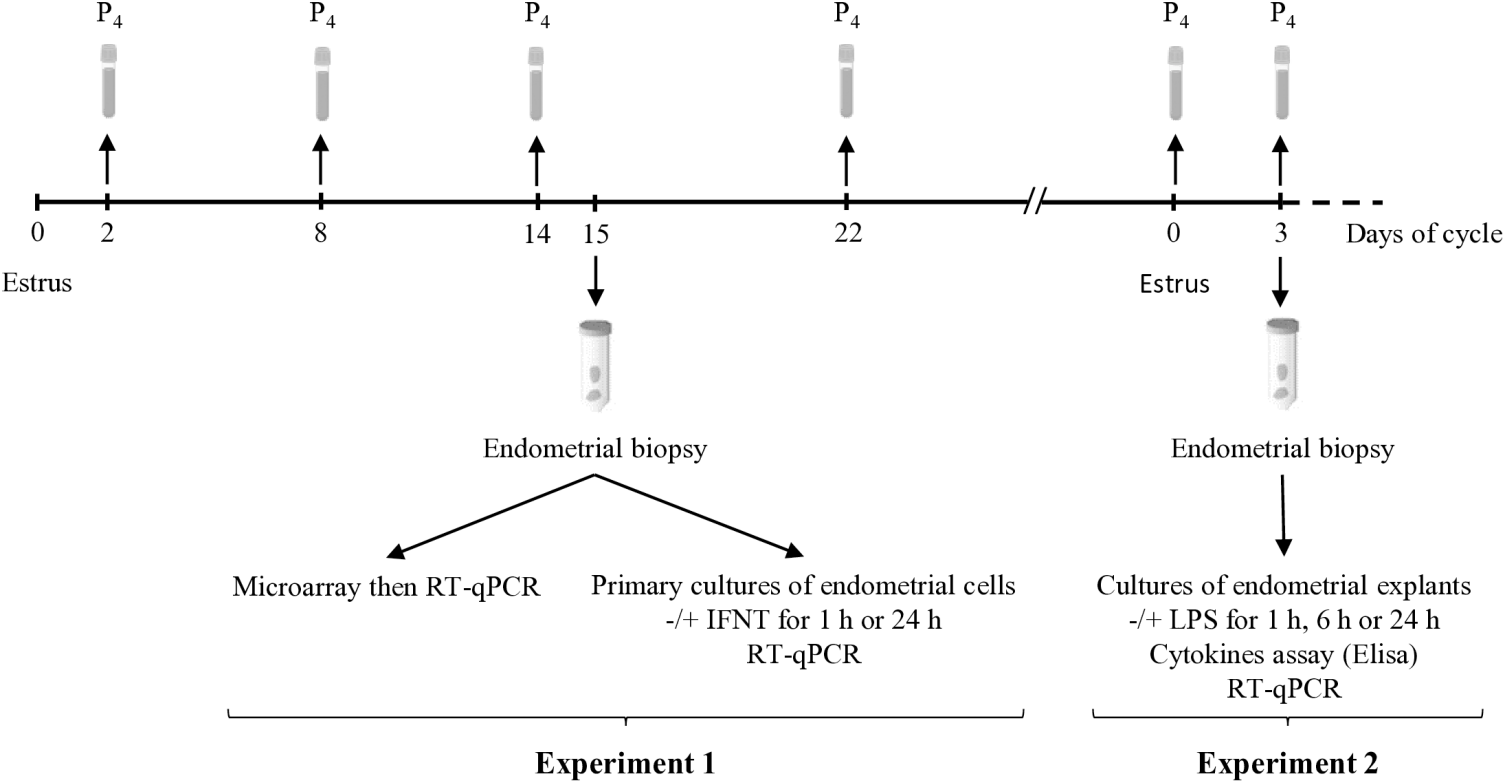
Experimental layout. After hormonal synchronization, endometrial biopsies were sampled from young females (YF, n = 6) and old females (OF, n = 8) at Day 15 of the estrous cycle for experiment 1 (Exp. 1) and on YF (n = 5) and OF (n = 5) at Day 3 for experiment 2 (Exp. 2). For determining P4 levels, blood samples were collected on Days 2, 8, 14, and 22 (Exp. 1) and on Days 0 (estrus) and 3 (Exp. 2).

To confirm the stage of the estrous cycle, serum progesterone (P4) concentrations were measured. Blood was collected on Days 2, 8, 14, and 22 of the estrous cycle for experiment 1 (6 YF and 8 OF) and on Days 0 and 3 for experiment 2 (5 YF and 4 OF; Fig 1). After centrifugation, serum was isolated and P4 concentration was analysed using an Enzyme-Linked Immunosorbent ELISA assay [28]. The intra-assay coefficient of variation of the quality control at a concentration of 1 ng/mL was 7.2%.

Endometrial biopsy samples were collected, under peridural anaesthesia (Procamidor ®, France) using a Kevorkian-Younge uterine biopsy forceps (Alcyon, Paris, France) as previously described [9]. The biopsies were performed only after confirming the effectiveness of the anaesthesia, which was assessed by observing tail relaxation and flaccidity. All manipulations were performed in a calm environment by a certified experimenter using precise and controlled techniques. For each female, 2–3 biopsies were taken from the middle of each uterine horn – ipsilateral and contralateral to the corpus luteum. A systematic macroscopic examination of the biopsies allowed for the careful identification and exclusion of caruncular tissue. Only intercaruncular tissue was retained and used for the analyses. Biopsies were taken on Day 15 from 6 YF and 8 OF (4–5 years and 13–14 years, respectively; experiment 1) and on Day 3 from 5 YF and 5 OF (6–7 years and 14–15 years, respectively; experiment 2).

### Total RNA preparation

Total RNA was extracted from biopsies, endometrial explant cultures or primary cultures of glandular epithelial or stromal cells using TRIzol Reagent (ThermoFisher Scientific) as previously described by [29] and purified on Qiagen columns (RNeasy Mini kit; Qiagen, Courtaboeuf, France) according to the manufacturer’s recommendations. Total RNA was quantified using Nanodrop ND-3300 spectrophotometer (Labtech, Wilmington, DE, USA). The RNA integrity (RIN) was evaluated using the Agilent 2100 ® Bioanalyzer (@bridge ICE -Iso Cell Express-, INRA, France: https://www6.jouy.inra.fr/ice). Only total RNA samples with a RIN ranged from 7 to 9.5 were conserved and stored at −80°C until use (array hybridization, microfluidic analyses and reverse transcription polymerase chain reaction (RT-PCR) quantification).

### Experiment 1: Impact of ageing on the bovine endometrium at Day 15 post-estrus

#### Microarray hybridization.

Transcriptomic analyses were performed using total RNA extracted from endometrial biopsies collected on D15 from the uterine horn ipsilateral to the corpus luteum, obtained from 4 YF and 4 OF (Fig 1; S1 Table). Gene expression profiles were determined using a custom bovine array, spotted with 61,325 probes that represented 23,926 unique transcripts, as described previously [30]. More than ninety-seven percent of the transcripts on the array are derived from the *Bos taurus* genome (taxid:9913; genome assembly UMD3.1) and annotated by Ensembl (http://www.ensembl.org/Bos_taurus/Info/Index). The remaining 3% are control elements (technical spike-ins, synthetic sequences and endogenous reference controls).

Cyanine-3 (Cy-3) labeled cRNAs were prepared using 25 ng of total RNA with the One-Color Low Input Quick Amp Labeling kit (Agilent Technologies). Specific activities and cRNA yields were determined using the NanoDrop ND-1000 (Thermo Fisher Scientific). For each sample 600 ng of Cy3 labeled cRNA (specific activity > 6.0 pmol of Cy3/μg of cRNA) were fragmented at 60°C for 30 min and then hybridized to the custom bovine arrays for 17 h at 65°C following the manufacturer’s instructions (Agilent Technologies) as described in [30]. The microarray data were submitted to GEO (Gene Expression Omnibus) database (accession number GSE291161).

#### Microarray data analysis.

Differentially expressed genes (DEG) between OF and YF were identified using the FCROS (Fold Change Rank Ordering Statistics) method [31]. Transcripts were considered as differentially expressed when they exhibited a fold change |FC| ≥ 1.5 and a f-value ≤ 5%.

To determine the major biological functions and canonical pathways associated with the DEG, interaction networks or pathways were analyzed using Ingenuity Pathways Analysis (IPA) software (https://www.ingenuity.com). For the IPA analyses, the p-value was adjusted using Benjamini-Hochberg method (B-H p-value) with significance set at B-H p-value ≤ 0.05 and a Z-score ≥ 2 or ≤ −2.

#### Gene candidat approach: validation of microarray data.

To validate the transcriptome findings, expression levels of a selection of candidate genes were quantified via RT-qPCR using endometrial biopsies from 6 YF and 7 OF (S1 Table). The OF with a P4 level of 0.4 ng/mL on the 14th day of the estrous cycle was excluded from this analysis. Candidate genes were selected based on their high differential expression in the transcriptomic analysis and their relevance to uterine mediated fertility or ageing in the endometrium.

Total RNA (1 µg per sample) was reverse-transcribed into complementary DNA (cDNA) using 1 µL of Oligo(dT)_12–18_ primers at 0.5 µg/µL (Invitrogen, France), 1 µL of 10 mM dNTP at 0.5 mM each (Thermo Fisher Scientific, France), and 1 µL of Maxima H Minus Reverse Transcriptase enzyme at 200 U/µL (Thermo Fisher Scientific, France) in a final reaction volume of 20 µL, following the manufacturer’s instructions (Thermo Fisher Scientific, Les Ulis, France). Specific primer pairs for each candidate gene were designed using NCBI Primer BLAST (https://www.ncbi.nlm.nih.gov/tools/primer-blast/) and are presented in S2 Table. All primers were validated prior to use by sequencing the corresponding amplification products to confirm specificity. The efficiency of the primers was validated using a standard curve. For each primer pair, a series of 6 successive dilutions of cDNA, prepared from the pooled samples, was performed. Each dilution was analysed in duplicate to ensure reproducibility. An amplification curve was then generated to calculate the efficiency based on the variation in the threshold cycle (Ct) for each dilution point. The efficiencies obtained ranged from 81.66% to 115.39%, with an average efficiency of 91.33%.

Quantitative PCR (qPCR) reactions were then performed using 1 µg of cDNA, 15 µM primers, and 7.5 µL of SYBR Green real-time PCR master mix (Applied Biosystems, France) using StepOnePlus Real-Time PCR Systems (Thermo Fisher Scientific, Les Ulis, France). All PCR reactions were performed in duplicate, in a final reaction volume of 15 µL, with RNase/DNase-free water. The amplification conditions were as follows: initial denaturation of 95°C for 10 minutes, followed by 45 cycles of denaturation at 95°C for 15 seconds, annealing at 60°C for 60 seconds, and DNA elongation at 72°C for 40 seconds. Product specificity was evaluated by analysing the melting curves and performing sequencing of the amplicons. A standard curve was included for each gene to generate the arbitrary expression values for the candidate genes [32]. A duplicate NTC (No Template Control) was included for each gene to assess potential contamination. A separate plate was used for each gene. Amplification products for each gene were sequenced to verify their specificity.

Normalization of candidate mRNA expression levels was achieved using the three most stable reference genes (*C2ORF29*, *RPL19*, and *SLC30A6*) from six tested reference genes, as determined by qBasePlus software (Biogazelle, Zwijnaarde, Belgium). The relative expression level of each gene was reported as mean calibrated normalized relative quantity (CNRQ) values in arbitrary units.

#### Isolation and primary culture of bovine endometrial cells.

For cell culture, endometrial biopsies collected from the ipsilateral and contralateral uterine horns of 3 OF and 3 YF at D15 were used (S1 Table). Immediately after collection, biopsies were immersed in 15 mL of phosphate-buffered saline (PBS) with Ca^2^⁺ and Mg^2^⁺ (Sigma-Aldrich, France) and transported on ice (4°C) under sterile conditions to the laboratory within approximately 3 hours.

Upon arrival, samples were rinsed twice with 15 mL of PBS (with Ca^2^⁺ and Mg^2^⁺) to remove blood residues. Biopsies were then blotted dry and placed in sterile Petri dishes. For each female, approximately 0.5 g of tissue was manually minced using a scalpel under sterile conditions in a laminar flow hood until reduced to a fine tissue homogenate. The minced tissue was enzymatically digested in 10 mL of a solution containing collagenase (C5138; 500 IU/ml; Sigma-Aldrich, France) and hyaluronidase (H3506; 250 IU/ml; Sigma-Aldrich, France) prepared in Euroflush medium (IMV 19450, France). Samples were incubated for 1.5 hours at 37°C in a water bath. The resulting cell suspension was diluted in 20 mL of Euroflush medium, transferred to 50 mL Falcon tubes, and centrifuged at 1,000 rpm for 10 minutes. The cell pellet was resuspended in 30 mL of complete medium consisting of DMEM-F12 with 15 mM HEPES (Gibco, France) supplemented with 10% fetal bovine serum (Gibco, France), 1% Glutamax (Sigma-Aldrich, France), 1% Penicillin–streptomycin (10 000 UI – 10 mg; Sigma-Aldrich, France), 1% Insuline-transferrine-sélénium (ITS 1 mg – 0.55 mg – 0.5 µg; Gibco, France), 1% Amphotericin B (10 mg/mL; Biosera, France), 1% gentamycin (10 mg/mL; Biosera, France), 1% Nystatin (100 000 UI/mL; Sigma-Aldrich, France)..

To remove undigested tissue and collagen fibres, the cellular suspension was first filtered through a 100 µm cell strainer. To separate glandular epithelial and stromal cells, a second filtration was then performed using a 30 µm filter (pluriStrainer, France). Glands were retained on the 30 µm filter, while stromal cells passed through and were subsequently filtered through a 10 µm filter (PluriStrainer, France).

Glands were seeded in the complete medium at a density of 4 × 10⁶ cells per 90 mm cell culture dish (64 cm^2^ surface area) and incubated at 38°C under 5% CO₂ and humidified atmosphere. Stromal cells were seeded at 16 × 10⁶ cells per Primaria™ dish (Corning, France) and incubated at 38°C under 20% CO_2_ for 2 hours to specifically promote their adhesion to the plastic, thereby facilitating the selection of this cell type. The medium was changed three times to remove non-adherent cells. Adherent stromal cells were then cultured in complete medium at 38°C under 5% CO_2_ and humidified atmosphere.

For each cell type, the medium was renewed every 2–3 days. To ensure cell type purity, only epithelial cells that formed a continuous monolayer within two days were maintained in culture and were considered epithelial glandular cells. After 6–7 days, when cells reached 80–90% confluence, they were trypsinized using TryLE Express (Gibco-Life Technologies, France) and reseeded into 12-well plates, with each well having a diameter of 22 mm. Cells were seeded at densities of 2.3 × 10⁴ glandular epithelial cells and 1.2 × 10⁴ stromal cells per well, in 1 mL of complete medium.

#### IFNT treatment of primary bovine endometrial cells.

After five days of culture, pre-confluent cells (80–90% confluence) were incubated with a modified medium in which fetal bovine serum was replaced by 0.1% bovine serum albumin. Cells were then treated in the absence or presence of recombinant ovine IFNT (100 ng/mL; produced by G. Charpigny, INRAE, France) for either 1 hour or 24 hours. For each cell type (glandular epithelial cells and stromal cells), experiments were performed in triplicate using 12-well plates (TPP, Dutscher). For wells intended for 1-hour treatments, IFNT (or control medium) was added 1 hour before the end of the 24-hour incubation period.

At the end of the treatment, the culture medium was collected, and cells from each well were lysed in 200 µL of RLT Plus buffer (RNeasy Plus Mini Kit, Qiagen, France) and stored at –80 °C until RNA extraction.

Total RNA was extracted from each type of cultured endometrial cells as described above. Expression levels of 7 reference genes and 89 selected genes (S2 Table) were quantified using the BioMark HD high-throughput microfluidic qPCR technique (Fluidigm, Genomeast Platform, Strasbourg, France). These genes were selected based on their biological relevance to pregnancy establishment. Among the 89 selected genes, the abundance of 77 transcripts in glandular epithelial cells and 79 in stromal cells was accurately quantified.

Normalization of transcript levels was conducted using the two most stable reference genes (*PCNP* and *FRG1*), as determined by qBasePlus software (Biogazelle).

### Experiment 2: Impact of ageing on endometrial response to inflammation

Experiment 2 was conducted 21 months after experiment 1, using the available animals. Endometrial explants were collected from 5 YF and 5 OF on Day 3 of the estrous cycle (Fig 1 and S1 Table). For each animal, explants derived from biopsies collected from the uterine horns ipsilateral and contralateral to the corpus luteum were incubated in 1 mL of Roswell Park Memorial Institute (RPMI) medium (Sigma, France), supplemented with 1% Glutamax (200 mM L-alanyl-L-glutamine; Gibco, France), 1% penicillin-streptomycin (Sigma-Aldrich, France), 1% amphotericin B (Biosera, France) and 1% insulin-transferrin-selenium supplement (Gibco, France). Explants were incubated for 1h, 6h or 24h in the presence or absence of LPS (1 µg/mL; strain O111: B4; Sigma-Aldrich, France), at 38°C under 5% CO_2_ in humidified air. At the end of each incubation period, explants were removed from the medium, centrifuged to remove residual incubation fluid, weighed, then frozen in liquid nitrogen and stored at −80°C. Incubation media were also collected, centrifuged, aliquoted into 0.25 mL, frozen in liquid nitrogen, and stored at −80°C pending analysis of cytokine concentration.

#### Quantification of transcript levels by microfluidic qPCR.

Total RNA was extracted from endometrial explants after LPS challenge. Due to insufficient mRNA quantity for one of the OF, the expression of 89 genes of interest and 7 reference genes (S2 Table) was quantified from the mRNA extracted from 5 YF and 4 OF (S1 Table) using the BioMark HD high-throughput microfluidic qPCR approach (INRAE, Nouzilly). Of the 89 candidate genes tested, the expression levels of 82 genes were accurately quantified. Normalization of transcript levels was performed using the two most stable reference genes (*C2ORF29* and *SLC30A6*) as determined by qBasePlus software (Biogazelle).

#### Quantification of cytokines concentration in incubation media.

The culture medium from explants from 5 YF and 4 OF was collected after 24 hours of culture in the presence or absence of LPS to assess cytokine assays. To account for basal cytokine levels, control samples consisting of culture medium incubated for 24 hours without explants were included under both LPS-treated and untreated conditions. A custom Milliplex cytokine assay based on Luminex technology specifically developed for ruminants (MERCK-Millipore, Custom bovine/ovine assay 1.0; G. Foucras, INRAE-ENVT, Toulouse; [33]) was used to quantify 14 cytokines (IL-1α, IL-1β, IL-2, IL-4, IL-6, IL-10, IL-17, CCL2, CCL3, CCL4, CCL5, CXCL10, IFN-γ and TNF-α).

### Statistical analysis

P4 concentrations and RT-qPCR quantification of endometrial transcript levels were compared between OF and YF using a Mann-Whitney test (p ≤ 0.05), conducted with GraphPad Prism 9.0.2.

To evaluate the effects of recombinant IFNT treatment and ageing on primary cell cultures of glandular or stromal origins, candidate transcript levels were analysed between OF and YF using a two-way ANOVA performed in R software (p ≤ 0.05). For the endometrial explants derived from OF and YF, a two-way ANOVA was also performed with R software (p ≤ 0.05) to assess the effect of LPS treatment and ageing on expression levels of candidate genes and cytokine concentrations present in the culture media.

The data are presented as mean ± SEM (standard error of the mean).

## Results

### Experiment 1: Impact of ageing on endometrial function at D15

#### Progesterone serum.

P4 concentrations showed no significant difference between the young and old females sampled on Days 2, 8, 14 and 22 of the estrous cycle (S1 Fig). One old female did not exhibit cyclic activity (P4 concentration was 0.4 ng/mL on the 14th day of the estrous cycle).

#### Ageing impacts endometrial transcriptional profiling.

Using total RNA, transcriptomic analyses and the validation of a selection of DEG by RT-qPCR included cycling females, with P4 levels ranging from 4.4 to 7.6 ng/ml on Day 14 of the estrous cycle (S2 Fig). Transcriptomic analyses were performed with 4 females per group, then RT-qPCR confirmation was performed with 6 YF and 7 OF.

**Fig 2:**
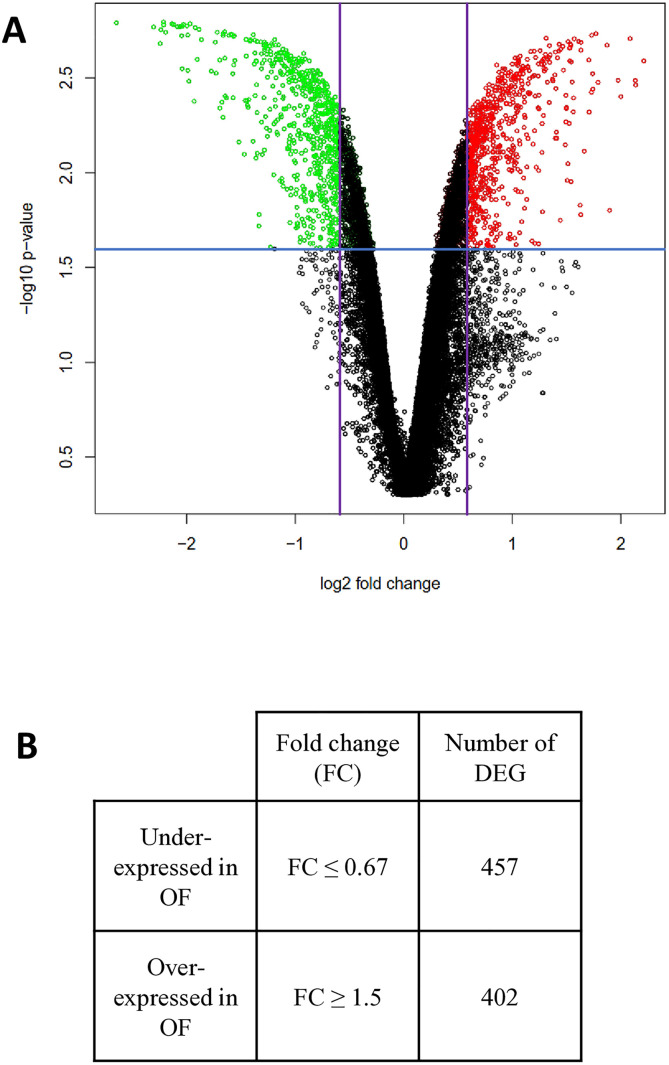
Results of microarray data in experiment 1. Transcriptomic analyses were performed using total RNA extracted from endometrial biopsies collected on Day 15 of the estrous cycle from old females (OF, n = 4) and young females (YF, n = 4). Statistical analyses were performed using the F-CROS method. (A) Volcano-plot of microarray data. Each circle represents a probe. The blue horizontal line represents the error threshold set at 5%. The vertical purple lines indicate the threshold used (|FC| ≥ 1.5). The green and red circles correspond to transcripts significantly over- or under-expressed in old females, respectively. (B) Table of the differentially expressed genes (DEG) between old and young females. FC = OF/ YF; f-value ≥ 0.975 or f-value ≤ 0.025.

Using the fold change rank ordering statistics (FCROS) method, volcano plots and probabilities were used to visualize the distribution of DEG between OF and YF at Day 15 post-estrus (FC = OF/ YF; Fig 2). Eight hundred and fifty-nine (859) DEG were identified between OF and YF (f-value ≥ 0.975 or f-value ≤ 0.025; |FC| ≥ 1.5), of which 402 were over-expressed (f-value ≤ 0.025 and FC ≥ 1.5) and 457 were under-expressed in OF (f-value ≥ 0.975 and FC ≤ 1/1.5) compared with YF (Fig 2 and S3 Table). Among the 859 DEG, only 34 displayed a |FC| ≥ 3 (S3 Table).

Among the 402 DEG over-expressed in OF compared with YF, *ASB2* (FC = 4.94), *ACTG2* (FC = 4.73), *CCL21* (FC = 4.58), *DTNA* (FC = 4.39), *ACTA1* (FC = 3.95), and *OXTR* (FC = 3.78) were among the DEG, displaying the highest FC. On the other hand, the most under-expressed genes in OF compared with YF included *CLEC4E* (FC = 0.22) *IGFBP1* (FC = 0.27), *IGFBP2* (FC = 0.31), *COL4A4* (FC = 0.40), *C PA3* (FC = 0.43) and *COL4A3* (FC = 0.45).

In order to validate the microarray data, expression levels of a selection of DEG identified between OF and YF, relevant to uterine receptivity or ageing, were quantified by RT-qPCR (S3 Fig). As determined by the microarray analyses, RT-qPCR analyses confirmed that expression level of *DTNA* was significantly higher in OF than in YF (*P* ≤ 0.05) whereas transcripts abundance of *ADAMDEC1*, *CDH11*, *COL4A3*, *CPA3*, *RSAD2*, *SCARA5*, *SERPINA14*, *SMPDL3B* and *TFAP2C* (*P* ≤ 0.05) as well as *IGFBP1* and *IGFBP2 (P* < 0.01) were significantly lower in OF compared with YF.

#### Ageing is associated with specific canonical pathways in the endometrium.

**Fig 3:**
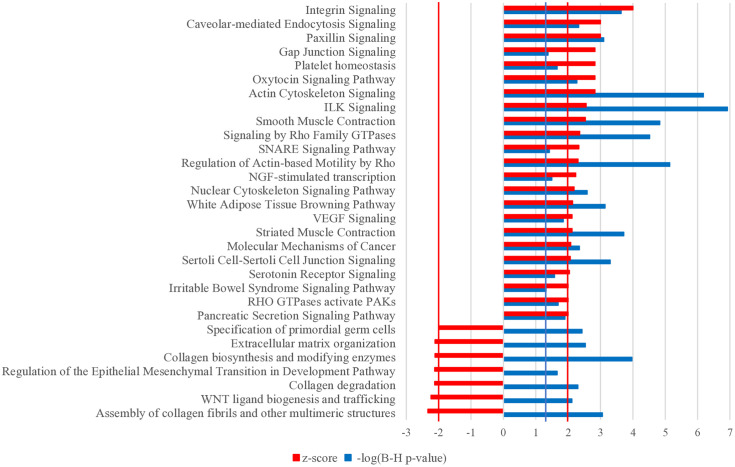
Canonical pathways associated with DEG identified in experiment 1. Significant predicted canonical pathways associated with DEG were identified from endometrial biopsies collected on day 15 of the estrous cycle in old females (OF, n = 4) compared to young females (YF, n = 4). Canonical pathways are considered significant when -log (B-H p-value) ≥ 1.301 (vertical blue line). Ingenuity Pathway Analysis (IPA) defines a canonical pathway as inhibited or activated in OF if the Z-score is ≤ −2 or ≥ 2 respectively (vertical red lines). Only the canonical pathways that are predicted to be activated or inhibited are presented and ranked in descending order of Z-score.

Out of the 859 DEG identified when the endometrium of OF was compared with YF, 798 were successfully mapped then 748 were subsequently analyzed using the IPA software. A significant association was established for 137 canonical pathways (B-H *p*-value ≤ 0.05), of which 23 were predicted to be activated (Z-score ≥ 2) and 7 inhibited (Z-score ≤ −2) in OF (Fig 3). The canonical pathways predicted to be inhibited in OF are mainly associated with the organisation and regulation of the extracellular matrix, such as “Assembly of collagen fibrils and other multimeric structures” (B-H p-value = 8.7E-04), “Collagen degradation” (B-H p-value = 5.0E-03), “Collagen biosynthesis and modifying enzymes” (B-H p-value = 1.1E-04) and “Extracellular matrix organization” (B-H p-value = 3.0E-03). Among the canonical pathways activated in OF, some are related to inflammation and immunity responses, such as “ILK signaling” (B-H p-value = 1.2E-07) and “Platelet homeostasis” (B-H p-value = 2.2E-02), while others are linked to regulation of cell motility and cytoskeleton such as the “RHO GTPases activate PAKs” (B-H p-value = 2.0E-02), “Regulation of Actin-based Motility by Rho” (B-H p-value = 7.2E-06) and “Actin cytoskeleton signaling” (B-H p-value = 6.6E-07). Canonical pathways associated with endometrial receptivity were also activated in OF, including the “Oxytocin signaling pathway” (B-H p-value = 5.4E-03), “VEGF signaling” (B-H p-value = 1.4E-02), and “Integrin signaling” (B-H p-value = 2.3E-04).

Out of the 241 significant (B-H p-value ≤ 1.6E-04) categories of functions identified by IPA (S5 Table), 14 biological functions were predicted to be increased (Z-score ≥ 2) and 12 were predicted to be decreased (Z-score ≤ −2) in OF (Table 1). Overall, movement of immune cells was strongly represented among the increased functions in OF, with for example “Cell movement of neutrophils” (*Z*-score = 2.6), “Cell movement of phagocytes” (*Z*-score = 2.4), “Migration of granulocytes” (*Z*-score = 2.1) and “Cell movement of myeloid cells” (Z-score = 2.0). The functions predicted to be decreased in OF were primarily related to cell death and survival and cellular assembly and organization, such as “Development of benign tumor” (Z-score = −2.4), “Mature-B-cell neoplasm” (Z-score = −2.0), “Mature B cell malignant tumor” (Z-score = −2.0), as well as the regulation of cellular metabolism and signaling, such as “Calcinosis” (Z-score = −2.4), “Biosynthesis of cyclic nucleotides” (Z-score = −2.1) and “Metabolism of cyclic nucleotides” (Z-score = −2.1).

**Table 1:** Biological functions associated with DEG identified in experiment 1.

Diseases or Functions annotation	B-H p-value	Activation Z-score
Cell movement of neutrophils	9,1E-09	2,6
Female genital neoplasm	7,4E-31	2,4
Tumorigenesis of reproductive tract	5,4E-31	2,4
Cell movement of phagocytes	8,0E-10	2,4
Differentiation of epithelial tissue	2,7E-06	2,3
Ovarian tumor	6,1E-08	2,2
Contractility of smooth muscle	4,1E-05	2,2
Accumulation of leukocytes	7,6E-05	2,2
Recruitment of myeloid cells	2,0E-05	2,1
Migration of granulocytes	2,5E-05	2,1
Adhesion of immune cells	1,1E-06	2,1
Cell movement of myeloid cells	3,5E-12	2,0
Accumulation of cells	1,1E-04	2,0
Differentiation of epithelial cells	3,5E-06	2,0
Mature B cell malignant tumor	4,1E-08	−2,0
Mature B-cell neoplasm	3,6E-13	−2,0
Brain lesion	1,3E-33	−2,0
Biosynthesis of cyclic nucleotides	6,6E-05	−2,1
Metabolism of cyclic nucleotides	3,6E-05	−2,1
Non-astrocytoma brain tumor	2,1E-23	−2,2
Intracranial lesion	3,5E-32	−2,2
Brain tumor	3,3E-32	−2,2
Head and neck tumor	6,1E-57	−2,3
Calcinosis	2,1E-06	−2,4
Development of benign tumor	7,4E-09	−2,4
Benign solid tumor	3,0E-16	−2,5

Significant predicted biological functions associated with DEG were identified from endometrial biopsies collected on day 15 of the estrous cycle in old females (OF, n = 4) compared to young females (YF, n = 4). Biological functions are significant when B-H p-value ≤ 0.05 and are predicted by IPA to be decreased (Z-score ≤ −2) or increased (Z-score ≥ 2) in OF. Only the biological functions that are activated or inhibited are presented and ranked in descending order of Z-score.

#### Ageing is associated with specific networks and upstream regulators in the endometrium.

IPA network interaction analyses revealed 25 interaction networks globally linked to metabolism, immune response and inflammatory processes and cellular organization (S6 Table).

**Fig 4:**
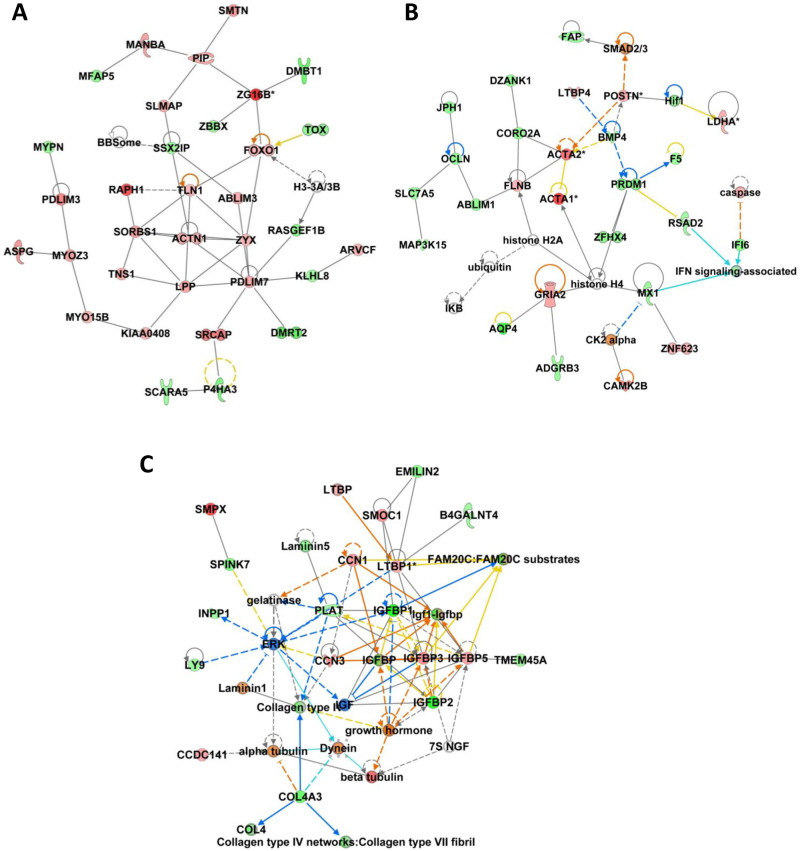
Major networks associated with DEG identified in experiment 1. Networks were generated from 859 differentially expressed genes (DEG) identified between old females (OF, n = 4) and young females (YF, n = 4) using IPA (Ingenuity Pathway analysis). The red node colour indicates up-regulated genes and the green color indicates down-regulated genes in OF versus YF endometrium. The colour intensity increases with the degree of fold change. Solid line indicates a direct interaction between nodes, and dashed line indicates an indirect relationship between nodes. (A) Network #1 – Cancer, Gastrointestinal Disease, Organismal Injury and Abnormalities. (B) Network #3 – Cancer, Organismal Injury and Abnormalities, Reproductive System Disease. (C) Network #15 – Cancer, Cellular Development, Cellular Movement.

Fig 4 illustrates three networks, namely « Cancer, Gastrointestinal Disease, Organismal Injury and Abnormalities », « Cancer, Organismal Injury and Abnormalities, Reproductive System Disease » and « Cancer, Cellular Development, Cellular Movement ». These networks include genes linked to cellular organization (*ACTA1*, *ACTA2*, *COL4A3*), metabolism (*IGFBP1*, *IGFBP2)* and interferon-stimulated genes (*MX1*, *RSAD2*), as well as *SCARA5*, whose expression is modulated by pregnancy in cattle. Most of these genes were validated by RT-qPCR.

IPA analyses identified 1526 significant upstream regulators (B-H p-value ≤ 0.05; S7 Table) of which 95 were predicted as activated (Z-score ≥ 2) and 51 were predicted as inhibited (Z-score ≤ −2) in OF (Table 2). Among the 10 most activated upstream regulators in OF, 4 were related to inflammatory processes (TGFB1, MAPK14, F3, Leukotriene D4). Among the 10 most inhibited upstream regulators in OF, several are related to the immune response (IFNAR, NC410, IRF3, IFNL1, IFN BETA).

**Table 2:** Top 10 activated or inhibited upstream regulators, associated with DEG identified in experiment 1.

Upstream Regulator	Molecule Type	Activation z-score	B-H p-value	Mechanistic Network
SRF	transcription regulator	4,9	2,0E-17	294 (18)
MRTFA	transcription regulator	4,5	9,2E-14	267 (18)
MRTFB	transcription regulator	3,9	2,4E-11	222 (14)
MYOCD	transcription regulator	3,9	1,9E-10	159 (10)
TGFB1	growth factor	3,7	3,1E-25	368 (22)
D-glucose	chemical – endogenous mammalian	3,6	6,0E-14	336 (26)
WWTR1	transcription regulator	3,3	1,7E-07	229 (15)
MAPK14	kinase	3,3	3,6E-04	285 (15)
F3	transmembrane receptor	3,2	3,6E-03	209 (15)
leukotriene D4	chemical – endogenous mammalian	2,9	4,0E-04	309 (21)
LATS (family)	Group	−2,7	1,1E-05	273 (13)
IFN BETA (family)	group	−2,7	1,2E-03	157 (13)
SIX1	transcription regulator	−2,7	5,7E-14	119 (7)
IFNL1	cytokine	−2,7	2,1E-02	289 (15)
AZD0156	chemical drug	−2,8	3,5E-06	
molybdenum disulfide	chemical reagent	−2,9	6,1E-13	
N-[N-(3,5-difluorophenacetyl-	chemical – protease inhibitor	−2,9	9,8E-03	282 (12)
L-Ala)]-S-phenylglycine t-butyl				
ester				
IRF3	transcription regulator	−3,0	1,7E-03	133 (15)
NC410	biologic drug	−3,1	1,3E-04	
IFNAR (family)	group	−3,4	6,3E-04	235 (16)

Significant predicted upstream regulators associated with DEG identified from endometrial biopsies taken on Day 15 of the estrous cycle in old females (OF, n = 4) compared to young females (YF, n = 4). Upstream regulators have been selected according to their significance (B-H p-value ≤ 0.05) and are predicted by IPA to be inhibited (Z-score ≤ −2) or activated (Z-score ≥ 2) in OF. The upstream regulators are ranked according to their Z-score.

#### Effect of ageing on the endometrial response to IFNT *in vitro.*

Among the 77 genes for which transcript levels were successfully quantified in primary cultures of glandular epithelial cells, 26 exhibited differential expression in response to ageing, incubation time, and IFNT treatment, whereas only 17 out of 79 quantified genes were differentially expressed in stromal cells. Ageing and IFNT response exhibited cell-type-specific effects (Table 3). In the primary cultures of glandular epithelial cells, after 1 hour of incubation, a marked effect of ageing and a limited effect of IFNT on the expression of candidate genes were observed, as reflected by the number of genes showing significantly different expression levels. Specifically, ageing significantly affected the expression of 5 genes (*ATP11C*, *CD3D*, *HEG1*, *SCN1A* and *SCRN3*) in the absence of IFNT (- IFNT) and 4 genes (*CAT*, *ELMO2*, *FNDC3B*, *SOD2*) in its presence (+ IFNT) and 7 genes (*BOLA-N*, *CYP7B1*, *FRG1*, *PCNP*, *PTGR1*, *S100A8*, *WARS*) commonly affected under both conditions. In contrast, in the presence of IFNT, the expression of *DMBT1* (P = 0.038), *SCN1A* (P = 0.048), and *SCRN3* (P = 0.003) is decreased in YF, while the expression of *ADAMDEC1* (P = 0.027) and *MX2* (P = 0.035) is increased. In OF, only two genes are affected by IFNT treatment: *MX2* (P = 3.5E-05) and *OAS1* (P = 0.037), both of which show increased expression in the presence of IFNT. Conversely, after 24 hours of incubation, the effect of ageing faded, affecting the expression of 3 genes (*AIF1*, *COL26A1*, *SCN1A*) in the absence of IFNT, 8 genes in its presence (*BOLA-N*, *CAT*, *CYP7B1*, *ELMO2*, *FNDC3B*, *PCNP*, *SOD2* and *WARS*) and 3 genes (*FRG1*, *PTGR1*, and *S100A8*) both in the absence and presence of IFNT. The effect of IFNT, meanwhile, became more pronounced, with 5 genes (*BOLA-N*, *IFI27L2*, *MX1*, *OAS1*, and *WARS*) commonly affected in both groups, 8 genes (*ATP11C*, *CAT, ELMO2*, *FRG1*, *IL15*, *PCNP*, *PTGR1*, and *TAC3*) specifically affected (P ≤ 0.05) in YF and *CYP7B1* (P = 0.01) specifically affected in OF (Table 3; S2 Fig).

**Table 3:** Response of endometrial cells to interferon tau (IFNT) in experiment 1.

		1 h				24 h			
		Ageing		IFNT treat.		Ageing		IFNT treat.	
	Gene symbol	- IFNT	+ IFNT	YF	OF	- IFNT	+ IFNT	YF	OF
Glandular epithelial cells	*ADAMDEC1*			1.98					
	*AIF1*								
	*ATP11C*							0.46	
	*BOLA-N*							3.89	3.06
	*CAT*							0.63	
	*CD3D*								
	*COL26A1*								
	*CYP7B1*								0.64
	*DMBT1*			0.80					
	*ELMO2*							2.18	
	*FNDC3B*								
	*FRG1*							1.19	
	*HEG1*								
	*IFI27L2*							0.52	0.52
	*IL15*							1.94	
	*MX1*							50.76	39.00
	*MX2*			7.44	2.06			ND	101.00
	*OAS1*				2.23			74	48
	*PCNP*							1.08	
	*PTGR1*							0.54	
	*S100A8*								
	*SCN1A*			0.48					
	*SCRN3*			0.96					
	*SOD2*								
	*TAC3*							4.96	
	*WARS*							5.71	4.47
Stromal cells	*ADAMDEC1*				1.28				
	*ATP11C*			1.21				0.36	0.34
	*BOLA-N*			7.43	13.17			32.74	36.18
	*CD3D*				0.50				0.36
	*CYP7B1*								0.72
	*ELMO2*							2.39	2.37
	*FNDC3B*								0.66
	*FRG1*							1.25	
	*IFI27L2*							0.42	0.40
	*IL15*							3.96	3.96
	*LOC109562134*							0.52	
	*MOG*							3.09	
	*MX1*				144.78			25686	
	*MX2*			ND				46140	15554333
	*OAS1*			42.17	35.39			2036	2021
	*PCNP*							0.92	
	*WARS*				1.34			7.18	7.78

Glandular epithelial cells and stromal cells from endometrial biopsies taken on Day 15 of the estrous cycle from young females (YF, n = 3) and old females (OF, n = 3) were cultured in the absence or presence of 100 ng/mL recombinant ovine interferon tau (IFNT) for 1 hour or 24 hours. Expression levels of candidate transcripts were quantified from total RNA using RT-qPCR. Two-way repeated-measures ANOVA test using R software was performed to identify significant differences according to ageing and IFNT treatment. Differences in transcript levels were considered statistically significant when P-value ≤ 0.05 (two-tailed). Significant differences are indicated by a coloured box background. For the IFNT treatment, the fold changes “+ IFNT/- IFNT” corresponds to the number indicated in each cell of the table (no significant difference). Only genes with significantly different expression levels for at least one condition are shown. Treat.: treatment. - IFNT: incubation in the absence of IFNT; + IFNT: incubation in the presence of IFNT. ND: not determined.

Ash color P ≤ 0.05.

Light black color P ≤ 0.01.

Dark black color P ≤ 0.001.

In the primary cultures of stromal cells, regardless of the incubation duration, ageing did not significantly (P > 0.05) affect the expression of candidate genes, with the exception of *ADAMDEC1,* which showed a significant difference (P = 0.007) after 1 or 24 hours of incubation with IFNT. However, after 1 hour of incubation, IFNT treatment led to a significant increase in the expression levels of *BOLA-N* and *OAS1* in both YF and OF. *ATP11C* expression increased significantly (P = 0.014) only in YF. In OF only, the expression levels of *ADAMDEC1* (P = 0.047), *MX1* (P = 0.022), and *WARS* (P = 0.019) increase significantly in presence of IFNT while *CD3D* expression decreased significantly (P = 0.006). Similar to the primary cultures of glandular epithelial cells, the effect of IFNT became more statistically significant in stromal cells after 24 hours of incubation. In details, IFNT treatment induced a significant increase in the expression levels of *BOLA-N*, *ELMO2*, *IL15*, *MX2*, *OAS1*, *WARS* and a significant decrease (p ≤ 0.05) in the expression of *ATP11C* and *IFI27L2* in both YF and OF. Additionnally, *CD3D* (P = 0.049), *CYP7B1* (P = 0.005) and *FNDC3B* (P = 0.005) expression was significantly decreased only in OF following IFNT treatment, whereas a significant (P = 0.005) decrease in PCNP expression and a significant increase in *FRG1* (P = 0.013), *MOG* (P = 0.009), *MX1* (P = 0.008) expression were observed only in YF following IFNT treatment.

The intensity of the response to IFNT, assessed by the FC (+IFNT/-IFNT) was not significantly (P > 0.05) different between OF and YF in each cell type.

### Experiment 2: Impact of ageing on endometrial response to inflammation

#### Determination of cycle stage by progesterone assay.

P4 levels at D3 were similar (p = 0.134) between the synchronised 6 YF and 3 OF. One old female was not synchronised.

#### Effect of ageing on endometrial response to LPS: transcript levels.

**Fig 5:**
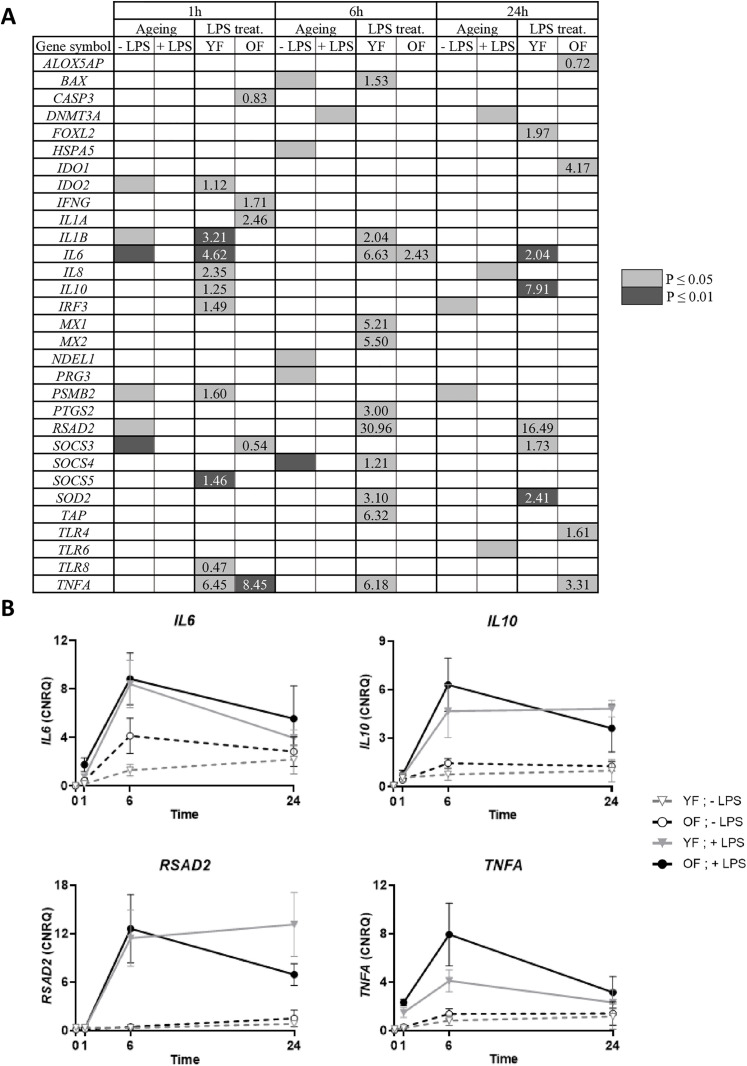
Response of endometrial explants to lipopolysaccharide (LPS) in experiment 2. Treat.: treatment. (B) Graphical representation of transcript levels for individual females after 24 hours incubation in the absence or presence of LPS. Mean + /- SEM. Explants were derived from endometrial biopsies sampled on Day 3 of the estrous cycle. Explants were cultured in the absence or presence of 1 μg/mL LPS for 1 hour, 6 hours or 24 hours. Young females (YF), n = 5; old females (OF), n = 4. (A) Effect of LPS and ageing on transcripts level. Expression levels of candidate transcripts were quantified on total RNA using microfluidic RT-qPCR. Two-way repeated-measures ANOVA test using R software was performed to identify significant differences according to ageing and LPS treatment. Differences in transcript levels were considered statistically significant when P-value ≤ 0.05 or lower (two-tailed). Significant differences are indicated by a coloured box background. For the LPS treatment, fold changes « + LPS/ - LPS » are indicated. Only genes with significantly different expression levels for at least one condition are shown.

As shown in Fig 5A, in the absence of LPS, 6 (*IDO2*, *IL1B*, *IL6*, *PSMB2*, *RSAD2*, *SOCS3*), 5 (*BAX*, *HSPA5*, *NDEL1*, *PRG3*, *SOCS4*) and 2 (*IRF3* and *PSMB2*) transcripts were differentially expressed between YF and OF (“Ageing”) after 1, 6 or 24 hours of incubation respectively. However, in the presence of LPS, the difference between YF and OF was no longer observed, as no candidate gene was differentially expressed between the two groups after 1 hour of treatment, only *DNMT3A* was differentially expressed after 6 hours and *DNMT3A*, *IL8* and *TLR6* after 24 hours of LPS exposure. Regardless of the incubation time (1, 6, and 24 hours), LPS treatment (“LPS treat.”) had a greater impact on YF than on OF, reflected by the number of transcripts whose expression levels were altered. After 1 hour of incubation, LPS treatment significantly affected the expression of 10 genes (*IDO2*, *IL1B*, *IL6*, *IL8*, *IL10*, *IRF3*, *PSMB2*, *SOCS5*, *TLR8* and *TNFA*) in YF, compared to 5 genes (*CASP3*, *IFNG*, *IL1A*, *SOCS3* and *TNFA*) in OF. After 6 hours, 11 genes (*BAX*, *IL1B*, *IL6*, *MX1*, *MX2*, *PTGS2*, *RSAD2*, *SOCS4*, *SOD2*, *TAP*, *TNFA*) were significantly upregulated in YF, whereas only *IL6* was upregulated in OF. Finally, after 24 hours of incubation, 6 genes (*FOXL2*, *IL6*, *IL10*, *RSAD2*, *SOCS3*, *SOD2*) were upregulated in YF, while only 4 genes (*ALOX5AP*, *IDO1*, *TLR4* and *TNFA*) showed significantly altered expression in OF. The intensity of the response, as assessed by the FC (+ LPS/ - LPS), was generally greater in YF than in OF for each incubation time.

#### Effect of ageing on endometrial response to LPS: cytokine concentration in the explant incubation medium.

**Fig 6:**
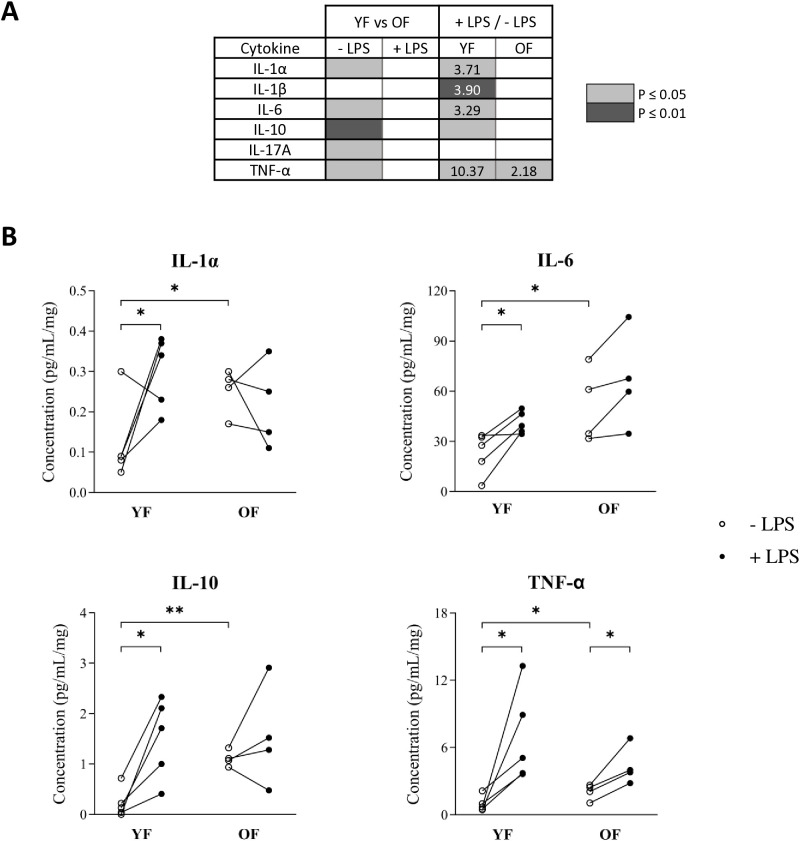
Effect of LPS on cytokine concentrations in the culture medium of endometrial explant in experiment 2. Explants were derived from endometrial biopsies sampled on Day 3 of the estrous cycle and were cultured in the absence or presence of 1 μg/mL LPS for 24 hours. Young females (YF), n = 5; old females (OF), n = 4. (A) Effect of LPS and ageing on cytokine concentration in the incubation medium. P-value was determined by an ANOVA test. Differences in cytokine levels were considered statistically significant when P-value ≤ 0.05 (two-tailed). Only cytokines with significantly different concentrations for at least one condition are shown. (B) Graphical representation of cytokine concentrations for individual females after 24 hours of incubation in the absence or presence of LPS. The cytokine concentration is expressed in picograms (pg) per milliliter of culture medium (mL), normalized to the weight of the explant tissue in milligrams (mg).

As observed for the transcripts, after 24 hours of incubation in the absence of LPS, ageing significantly influenced the concentrations of five cytokines quantified in the culture media of endometrial explants. However, in the presence of LPS, the concentrations of the 14 cytokines measured in the culture medium of endometrial explants were similar between YF and OF (Fig 6).

LPS treatment significantly affected the concentration of 5 cytokines in YF and only one in OF (Fig 6A). Indeed, in YF, LPS induced a significant increase in the concentration of pro-inflammatory cytokines IL-1α, IL-1β, IL-6, and the anti-inflammatory cytokine IL-10 (FC ranging from 3.6 to 5.0). LPS treatment significantly increased the concentration of the pro-inflammatory cytokine TNF-α in both YF and OF, with no significant difference in fold change between the two groups (Fig 6B).

Ageing or LPS treatment had no significant effect on the concentration of the pro-inflammatory cytokines such as CCL2, CCL3, CCL4, CXCL10, IFN-γ, and IL-2, and the anti-inflammatory cytokines such as IL-1RA and IL-4.

## Discussion

In mammalian females, research has primarily focused on the overall impact of age on pregnancy outcomes following mating or artificial insemination [34]. While studies have documented age-related changes in ovarian phenotypes, oocyte quality, and the hypothalamic-pituitary axis in both human and animal models, the specific effects of ageing on the endometrium—at both molecular and functional levels—have been explored in a limited number of studies.

The bovine model is frequently cited in the study of female reproductive function, including the effects of ageing [35–37]. More specifically, cloned cattle raised in experimental farms provide a relevant model, as their use minimises genetic and environmental variability. The use of cloned young (3 years old) and old (10 years old) cows was published as an experimental model for investigating the effect of aging on ovarian response to stimulation, using a protocol designed for women [38]. Very few laboratories have been able to derive such an experimental model. Our INRAE model of bovine somatic clones, that involves young primiparous females and old nulliparous females, has offered the unique opportunity to decipher the molecular and biological effect of ageing on the endometrium.

In our experiments, P4 level was similar between the two experimental groups during the luteal phase. These results are consistent with previous studies conducted on bovine females, one comparing P4 levels in cows aged 13–14 with their daughters aged 1–4 years, and the other one comparing cows aged 16–17 years with cows aged 4–5 years [35,39]. A third study presented some nuances: the P4 concentration in young females (2.9 years) is significantly lower on both the 7th and 12th days of the cycle compared to that in older females (15.7 years). However, no difference was observed between the middle-aged group (6.7 years) and the older cows [36]. This latter comparison closely matches the age profile of the females involved in our study. The exact parity of the cows in these studies, however, was not specified. Given the established association between early luteal phase P4 levels and the likelihood of a successful pregnancy, we have therefore considered that P4 secretion alone does not account for the decline in fertility associated with age [40].

In our experimental bovine animal model with a fixed genotype, our study also showed that ageing was associated with variations in gene profiles of the endometrium at D15.

Among the 859 DEG we found between YF and OF, a limited number displayed a |FC| ≥ 3. This limited fold change is consistent with previous observations published for human endometrium, with 28 endometrial genes found to be differentially expressed with a fold change greater than 3 between women younger or older than 35 [41]. Interestingly, our RT-qPCR analyses confirmed that *CDH11*, *SCARA5*, *SMPDL3B*, and *TFAP2C*, on the one hand, and *DTNA*, on the other hand, were respectively under-expressed and over-expressed in OF. These findings are similar to those observed in women, namely, a under-expression of *CDH11* and *SCARA5* in senescent decidual cells, a under-expression of *SMPDL3B* and *TFAP2C*, and an over-expression of *DTNA* in the endometrium of women over 35 years old [41,42]. This striking similarity in gene regulation between humans and cattle—species that differ in endometrial cell composition during the luteal phase—underscores the need for a comprehensive investigation into the biological roles of these genes in mammalian endometrial ageing.

In addition to the regulation observed in the senescence of decidual cells in women, *SCARA5* has also been identified as a differentially expressed gene in the endometrium of heifers with distinct fertility profiles. On days 7 and 14 of the estrous cycle preceding embryo transfer, *SCARA5* is underexpressed in the endometrium of heifers in which the transfer fails, compared to those that become pregnant [43]. Given its well-established role in humans as a tumor suppressor gene involved in limiting cell proliferation and promoting apoptosis, the decreased expression of *SCARA5* in the endometrium of old females may indicate a dysregulation of endometrial cell turnover, which could impair endometrial function and contribute to the reduced fertility associated with uterine ageing [44,45].

In our study, *CLEC4E*-the gene encoding the MINCLE protein, which is expressed on the surface of immune cells such as macrophages, dendritic cells, and natural killer cells and recognizes pathogen-associated molecular patterns (PAMPs) as well as damage-associated molecular patterns (DAMPs)-was the gene that presented the lowest transcript levels in the endometrium of OF compared with YF [46]. *CLEC4E* is also one of the most under-expressed genes during the mid-luteal phase in the endometrium of women with recurrent implantation failure. Additionally, in these women, the expression of *SIX1* was down-regulated [47]. This transcription factor has also been implicated in endometrial carcinogenesis, although the precise mechanisms through which it contributes to this process remain to be clarified [48]. Notably, SIX1, which regulates numerous genes involved in cell proliferation, migration, and senescence, is one of the most inhibited upstream regulators in OF [49]. These findings may impair tissue homeostasis and thereby contribute to the increased incidence of embryonic mortality observed in aging females [34]. In the OF group, our data underlined the reduced transcriptional expression of genes including *ADAMDEC1*, *CPA3*, *IGFBP1*, *IGFBP2, SCARA5* and *TFAP2C* involved in proliferative and metabolic processes, such as “Female genital neoplasm”, “Tumorigenesis of reproductive tract”, “Ovarian tumor”, “Benign solid tumor”. *IGFBP1* and *IGFBP2*, two members of the insulin-like growth factor binding protein family that modulate IGF activity and thereby influence cell proliferation, differentiation and survival, are known to be involved in the regulation of endometrial receptivity or decidualization [50,51]. *IGFBP1* transcript levels are lower in the endometrium of sub-fertile heifers compared with highly fertile ones, a result similar to what has been observed in the endometrium of infertile women compared with fertile ones [52,53]. *In vitro*, *IGFBP1*, a marker of endometrial decidualization in women, is less expressed in the stromal endometrial cells of women over 36 years old compared with those under 36 [54]. On day 17 of the estrous cycle, *IGFBP2* expression was lower in the endometrium of infertile heifers compared with sub-fertile ones [1]. CPA3 encodes an enzyme that is mainly expressed by mast cells, a type of immune cell found in the endometrium. The number and activity of endometrial mast cells fluctuate throughout the menstrual cycle, with a specifically increased presence and activation reported in women experiencing recurrent pregnancy loss [55]. The higher *CPA3* expression observed in the endometrium of young mice compared to older ones may reflect age-related changes in immune cell function and tissue homeostasis, a finding that is also confirmed in our study, where the endometrium of young females shows higher *CPA3* expression that of older females [56]. These findings suggest a deregulation of the metabolic, immune and proliferative processes in the endometrium associated with age, that may affect endometrial receptivity and the ability of this tissue to interact with the implanting conceptus.

Furthermore, *COL4A3* and *COL4A4* are among the most under-expressed genes in OF. These genes are involved part of canonical pathways that are inhibited in OF, including “Assembly of collagen fibrils and other multimeric structures,” “Collagen degradation,” “Collagen biosynthesis and modifying enzymes,” and “Extracellular matrix organization,” suggesting an alteration in the structure and function of the extracellular matrix of the endometrium in older females.

In the OF group, *CCL21* transcript levels were higher than those quantified in the YF group and *CCL21* was involved in biological pathways related to immune cell trafficking which were activated in females from the OF group. This gene, whose expression varies depending on the estrous cycle or pregnancy status in cows and sows, was reported to be over-expressed in the luminal epithelial cells of the endometrium in cows with persistent subclinical endometritis, compared to those that are healthy or have resolved the condition [57–59]. Indeed, CCL21 is a chemokine notably expressed by stromal cells. It has a high affinity for the CCR7 receptor, which is expressed by various immune cells, including naïve T and B cells, central memory T cells, dendritic cells, and natural killer cells. During an inflammatory process, CCL21 expression can be upregulated, thereby promoting the chemotactic recruitment of these immune cells to lymphoid or inflamed tissues [60]. Overall, the increased expression of *CCL21* in OF suggests an age-associated alteration in endometrial immune regulation. This may reflect a heightened inflammatory state or a dysregulated immune environment in the ageing uterus. Biological functions related to immunity and immune cell migration are also predicted to be activated in our model of OF. Our results are consistent with the activation of age-related inflammation pathways previously described in bovine and murine endometrial cells [56,61]. Collectively, these results support the emergence of an inflammatory phenomenon in the ageing endometrium.

In our model of endometrial explants incubated with LPS, our results showed that explants from YF are more sensitive to LPS treatment than those from OF, when transcript and protein levels of a selection of cytokines were quantified. Our results are consistent with findings from studies that examined cytokine production in bovine endometrial explants or cells, collected at various stages of the estrous cycle and cultured for up to 48 hours with increasing concentrations of LPS, including 1 µg/mL [62,63]. In contrast, in the OF group, cytokine protein content in the culture supernatants remained unchanged after 24 hours in response to LPS. Our results suggest that the sensitivity of the bovine endometrium to an inflammatory trigger decreases with age. This could be explained by the chronic low-noise inflammation characteristic of ageing, or by a reduced capacity of the endometrium to respond to LPS challenge [64]. Further studies, with an extended incubation period and primary cultures of various endometrial cell types, could help clarifying whether the response of endometrial cells from old females is indeed reduced or simply delayed.

Exacerbated inflammation in the endometrium significantly compromises the establishment of gestation in human [65]. Our findings show that ageing is associated with molecular changes of the endometrium, that affect the sensor properties of the endometrium and its ability to respond to the implanting conceptus. These results are consistent with observations in alpacas, a species in which females over 15 years of age exhibit lower birth rates following embryo transfer compared to those under 15 years of age [19]. In our study, incubation of IFNT with endometrial cells revealed an altered response of ageing cells to this major factor of pregnancy recognition in ruminants. Our findings also demonstrated a cell-type-specific response to IFNT in term of transcript levels in YF and OF cells. Glandular epithelial cells exhibited a greater sensitivity than stromal cells to IFNT. These results are consistent with the cell-type-specific transcriptomic signatures previously demonstrated in the absence or presence of IFNT [66–68].

After 24 hours of incubation, glandular epithelial cells derived from YF endometrial biopsies appeared more sensitive to IFNT stimulation than those derived from OF, as indicated by a greater number of differentially expressed genes and stronger statistical significance. These data are consistent with the inhibition of the upstream regulator IFNAR in OF observed in our transcriptomic analyses. Previous studies have demonstrated that endometrial glands are crucial for the establishment of pregnancy in ruminants [69]. Consequently, the altered ability of the glandular epithelial cells to response to IFNT could interfere with pregnancy establishment, as suggested by Taniwaka and colleagues [61]. In their study, endometrial cells from young (3.8 years) and old (14.4 years) cows, regardless of cell type and parity, were cultured with IFNT, and the endometrial cells from older cows showed a reduced sensitivity to IFNT compared to those from younger cows [61]. Pregnancy failures reported in aged females may result from a compromised sensor property of the endometrial cells that affect the ability of this tissue to respond optimally to the signals emitted by the conceptus during the implantation process.

In our study, all OF were nulliparous, and all YF were primiparous. Therefore, the contribution of primiparity in the interpretation of our analyses cannot be ruled out. To our knowledge, limited information is available regarding the effect of parity on endometrial function. In cows, the pregnancy rate following an embryo transfer decreases as the recipient’s parity increases [20]. In this study parity and age were confounded. A study in aging female rats revealed that age, combined with parity and estrous cyclicity, significantly affects fertility in older rats. Specifically, the age-related decline in fertility is further exacerbated by increased parity, as multiparous females and those with disrupted estrous cyclicity are less likely to conceive with advancing age [70]. Furthermore, it has been shown that parity can induce a chronic inflammatory response. Indeed, Urzua *et al.* demonstrated that parity influences the systemic inflammatory response associated with the spread of ovarian cancer in aging mice, with multiparous mice exhibiting a more pronounced systemic inflammatory response compared to nulliparous mice [71]. An experimental model that includes young and old females with identical parity would refine our understanding of the effects specific for age and for parity on the endometrium.

In conclusion, our study has shown that ageing is associated with significant alterations in biological functions and molecular pathways that play critical roles for pregnancy establishment and progression in cattle. The consequences of ageing on pregnancy failures or prenatal programming of health in adult involve a skewed response of the endometrium to embryonic factors that deserve more detailed investigation. Given the specific physiological roles played by the various cell types of the endometrium, future investigations that address the impact of ageing on distinct endometrial cell types will bring new insights on the mechanisms underlying the alteration of the sensor and driver properties of the endometrium and their contribution to fertility and pregnancy outcomes [68,72]. Our current knowledge including this study prompts the need for addressing the negative effects of uterine ageing as part of fertility management in mammalian species including cattle and humans.

## Supporting information

S1 Fig Progesterone serum assay during the estrous cycle in experiment 1. Blood samples were collected on Days 2, 8, 14 and 22 of the estrous cycle on young females (YF, n = 6) and old females (OF, n = 7) included in the transcriptomic analyses and validation by RT-qPCR. Mean + /- SEM.
(TIF)

S2 Fig Individual serum progesteronemia during the cycle of experiment 1. Blood progesteronemia was measured at Days 2, 8, 14, and 22. For each young female (YF); For each old female (OF). Solid lines indicate females included in transcriptomic analyses and RT-qPCR confirmation (n = 4 per group). Dashed lines indicate additional females for RT-qPCR confirmation (n = 2 YF and n = 3 OF). In total, transcriptomic analyses were performed with 4 females per group, RT-qPCR confirmation was performed with 6 YF and 7 OF.
(TIF)

S3 Fig RT-qPCR validation of the microarray data in experiment 1. Expression levels of the transcripts were quantified by RT-qPCR using total RNA extracted from endometrial biopsies collected on Day 15 of the estrous cycle from 6 young females (YF, black bars) and 7 old females (OF, open bars). Expression levels for each gene were determined in calibrated normalized relative quantities (CNRQ) and were presented as mean + /- SEM. P value was determined by Mann-Whitney test. * P ≤ 0.05; ** P ≤ 0.01.
(TIF)

S4 Fig Response of primary cultures of endometrial cells to interferon tau (IFNT) in experiment 1. Principal component analyses were performed using the expression levels of 77 and 79 candidate genes quantified in 3 young females (YF) and 3 old females (OF) in glandular epithelial cells and stromal cells, respectively. Each point represents a female. Each asterisk represents the centroid of a group.
(TIF)

S1 Table Details of animals included in each experiment according to analyses performed. Exp. 1: experiment 1 (Day 15 of the estrous cycle); Exp. 2: experiment 2 (Day 3 of the estrous cycle).
(DOCX)

S2 Table Primers sequences used for RT-qPCR quantification of transcript levels. Microarray: validation of DEG identified by microarray.
(XLSX)

S3 Table Differentially expressed genes (DEG = 859) identified in endometrial biopsies collected at day 15 post-oestrus from old females (OF; n = 4) versus young females (YF; n = 4). FC: fold change. Fold change rank ordering statistics (FCROS) method (f-value ≤ 0.025 and ≥ 0.975; |FC| ≥ 1.5). f-value ≤ 0.025 and FC ≤ 0.67: DEG under-expressed in YF; over-expressed in OF. f-value ≥ 0.975 and FC ≥ 1.5: DEG over-expressed in YF, under-expressed in OF.
(XLSX)

S4 Table Ingenuity Canonical Pathways related to 859 DEG identified between old females (OF; n = 4) and young females (YF; n = 4) in endometrial biopsies collected on Day 15 post-oestrus. (XLS)

S5 Table Ingenuity Diseases or Functions Annotation related to 859 DEG identified between old females (OF; n = 4) and young females (YF; n = 4) in endometrial biopsies collected on Day 15 post-oestrus. (XLS)

S6 Table Networks related to 859 DEG identified between old females (OF; n = 4) and young females (YF; n = 4) in endometrial biopsies collected on Day 15 post-oestrus. (XLS)

S7 Table Upstream Regulator related to 859 DEG identified between old females (OF; n = 4) and young females (YF; n = 4) in endometrial biopsies collected on Day 15 post-oestrus. (XLS)

S8 Table Individual progesterone levels measured in Experiment 1 (Days 2, 8, 14 and 22 of the estrous cycle) and Experiment 2 (Days 0 and 3) in YF and OF. (XLSX)

S9 Table Individual RT-qPCR results from endometrial biopsies collected in Experiment 1. (XLSX)

S10 Table Individual RT-qPCR results on primary endometrial cells cultured for 1 hour or 24 hours in the presence or absence of IFNT in Experiment 1. (XLSX)

S11 Table Individual RT-qPCR results on endometrial explants cultured for 1, 6 or 24 hours in the presence or absence of LPS in Experiment 2. (XLSX)

S12 Table Cytokine concentrations in the incubation medium after 24h of incubation in the absence or presence of LPS. Cytokine concentrations in the incubation medium cultured with explant are expressed in pg/mL/mg. Explant mass in mg. The cytokine concentrations reported in the table were calculated as follows: (Cytokine concentration measured – Control cytokine concentration)/ Explant mass. For statistical analysis, negative values of IL-1RA, IL-17A, and IL-10 cytokine concentrations after correction were set to zero. Cytokine concentrations in the control incubation medium without explant are expressed in pg/mL.
(XLSX)

## References

[1] Moraes JGN, Behura SK, Geary TW, Hansen PJ, Neibergs HL, Spencer TE. Proceedings of the National Academy of Sciences of the United States of America. 2018;115(8). 10.1073/pnas.1721191115 PMC5828633 29432175

[2] Sánchez JM, Mathew DJ, Passaro C, Fair T, Lonergan P. Embryonic maternal interaction in cattle and its relationship with fertility. Reprod Domestic Animals. 2018;53(S2):20–7. 10.1111/rda.13297 30238655

[3] Neykova K, Tosto V, Giardina I, Tsibizova V, Vakrilov G. Endometrial receptivity and pregnancy outcome. J Matern Fetal Neonatal Med. 2022;35(13):2591–605. doi: 10.1080/14767058.2020.1787977 32744104

[4] Mansouri-Attia N, O S, Aubert J, Degrelle S, Everts RE, Giraud-Delville C. Endometrium as an early sensor of in vitro embryo manipulation technologies. Proc Natl Acad Sci USA. 2009;106(14):5687–92. 19297625 10.1073/pnas.0812722106 PMC2667091

[5] Macklon NS, Brosens JJ. The human endometrium as a sensor of embryo quality. Biol Reproduction. 2014;91(4). 10.1095/biolreprod.114.122846 25187529

[6] O S, Mansouri-Attia N, Lea RG. Novel aspects of endometrial function: a biological sensor of embryo quality and driver of pregnancy success. Reprod Fertil Dev. 2012;24(1):68. 10.1071/RD11908 22394719

[7] Woods L, Perez-Garcia V, Kieckbusch J, Wang X, DeMayo F, Colucci F, et al. Decidualisation and placentation defects are a major cause of age-related reproductive decline. Nat Commun. 2017;8(1):352. doi: 10.1038/s41467-017-00308-x 28874785 PMC5585348

[8] Wu J, Lin S, Huang P, Qiu L, Jiang Y, Zhang Y, et al. Maternal anxiety affects embryo implantation via impairing adrenergic receptor signaling in decidual cells. Commun Biol. 2022;5(1):840. doi: 10.1038/s42003-022-03694-1 35982177 PMC9388523

[9] Chankeaw W, Lignier S, Richard C, Ntallaris T, Raliou M, Guo Y, et al. Analysis of the transcriptome of bovine endometrial cells isolated by laser micro-dissection (2): impacts of post-partum negative energy balance on stromal, glandular and luminal epithelial cells. BMC Genomics. 2021;22(1):450. doi: 10.1186/s12864-021-07713-z 34139988 PMC8212477

[10] Cicinelli E, Vitagliano A, Loizzi V, De Ziegler D, Fanelli M, Bettocchi S. Altered gene expression encoding cytochines, grow factors and cell cycle regulators in the endometrium of women with chronic endometritis. Diagnostics. 2021;11(3):471. doi: 10.3390/diagnostics11030471 33800186 PMC7999985

[11] Murata H, Kunii H, Kusama K, Sakurai T, Bai H, Kawahara M. Heat stress induces oxidative stress and activates the KEAP1-NFE2L2-ARE pathway in bovine endometrial epithelial cells. Biol Reproduction. 2021;105(5):1114–25. 10.1093/biolre/ioab143 34296252

[12] Sant’Anna Monteiro da Silva E, Sanches Oquendo Júnior P, Gaspari Oquendo FM de, Stout TAE, de Ruijter-Villani M, Rodrigues TS, et al. Effect of duration of estradiol exposure on embryo survival and endometrial gene expression in anestrous embryo recipient mares. Theriogenology. 2024;226:1–9. doi: 10.1016/j.theriogenology.2024.05.039 38820771

[13] Talukder AK, McDonald M, Browne JA, Charpigny G, Rizos D, Lonergan P. Response of bovine endometrium to interferon tau in the presence of lipopolysaccharide. Theriogenology. 2024;229:169–77. doi: 10.1016/j.theriogenology.2024.08.026 39180888

[14] Zavatta A, Parisi F, Mandò C, Scaccabarozzi C, Savasi VM, Cetin I. Role of inflammaging on the reproductive function and pregnancy. Clinic Rev Allerg Immunol. 2022. 10.1007/s12016-021-08907-9 PMC8760119 35031955

[15] Briley SM, Jasti S, McCracken JM, Hornick JE, Fegley B, Pritchard MT. Reproductive age-associated fibrosis in the stroma of the mammalian ovary. Reproduction. 2016;:245–60. 27491879 10.1530/REP-16-0129 PMC4979755

[16] Ovadya Y, Landsberger T, Leins H, Vadai E, Gal H, Biran A, et al. Impaired immune surveillance accelerates accumulation of senescent cells and aging. Nat Commun. 2018;9(1):5435. doi: 10.1038/s41467-018-07825-3 30575733 PMC6303397

[17] Lee KA, Flores RR, Jang IH, Saathoff A, Robbins PD. Immune senescence, immunosenescence and aging. Front Aging. 2022;3:900028. 35821850 10.3389/fragi.2022.900028 PMC9261375

[18] Guo Y, Guan T, Shafiq K, Yu Q, Jiao X, Na D, et al. Mitochondrial dysfunction in aging. Ageing Res Rev. 2023;88:101955. doi: 10.1016/j.arr.2023.101955 37196864

[19] Vaughan J, Mihm M, Wittek T. Factors influencing embryo transfer success in alpacas—A retrospective study. Animal Reproduction Science. 2013;136(3):194–204. 23141430 10.1016/j.anireprosci.2012.10.010

[20] Ferraz PA, Burnley C, Karanja J, Viera-Neto A, Santos JEP, Chebel RC, et al. Factors affecting the success of a large embryo transfer program in Holstein cattle in a commercial herd in the southeast region of the United States. Theriogenology. 2016;86(7):1834–41. doi: 10.1016/j.theriogenology.2016.05.032 27364084

[21] Yamamoto T, Iwata H, Goto H, Shiratuki S, Tanaka H, Monji Y, et al. Effect of maternal age on the developmental competence and progression of nuclear maturation in bovine oocytes. Mol Reprod Dev. 2010;77(7):595–604. doi: 10.1002/mrd.21188 20575084

[22] Pasquariello R, Ermisch AF, Silva E, McCormick S, Logsdon D, Barfield JP. Alterations in oocyte mitochondrial number and function are related to spindle defects and occur with maternal aging in mice and humans†. Biol Reproduction. 2019;100(4):971–81. 10.1093/biolre/ioy248 30476005

[23] Zhang J-J, Liu X, Chen L, Zhang S, Zhang X, Hao C, et al. Advanced maternal age alters expression of maternal effect genes that are essential for human oocyte quality. Aging (Albany NY). 2020;12(4):3950–61. doi: 10.18632/aging.102864 32096767 PMC7066876

[24] Laser J, Lee P, Wei J-J. Cellular senescence in usual type uterine leiomyoma. Fertil Steril. 2010;93(6):2020–6. doi: 10.1016/j.fertnstert.2008.12.116 19217096

[25] Korzekwa AJ, Bah MM, Gęstwicka M, Socha B, Skarżyński DJ. Adenomyosis in the bovine uterus: correlation between frequency, age, and 17β-estradiol-progesterone equilibrium. Theriogenology. 2013;79(1):165–72. doi: 10.1016/j.theriogenology.2012.09.023 23122605

[26] Shrestha B, Paudyal S, Kaniyamattam K, Grohn YT. Organic dairy cattle longevity and economic implications: contemporary perspectives. J Dairy Sci. 2025. 10.3168/jds.2024-25767 39892593

[27] Ledoux D, Veissier I, Meunier B, Gelin V, Richard C, Kiefer H. Combining accelerometers and direct visual observations to detect sickness and pain in cows of different ages submitted to systemic inflammation. Sci Rep. 2023;13(1):1977. 36737469 10.1038/s41598-023-27884-x PMC9898231

[28] Canépa S, Lainé AL, Bluteau A, Fagu C, Flon C, Monniaux D. Validation d’une méthode immunoenzymatique pour le dosage de la progestérone dans le plasma des ovins et des bovins. Cah Techn Inra. 2008;64:19–30.

[29] Mansouri-Attia N, Aubert J, Reinaud P, Giraud-Delville C, Taghouti G, Galio L, et al. Gene expression profiles of bovine caruncular and intercaruncular endometrium at implantation. Physiological Genomics. 2009;39(1):14–27. 19622795 10.1152/physiolgenomics.90404.2008

[30] Carvalho AV, Canon E, Jouneau L, Archilla C, Laffont L, Moroldo M, et al. Different co-culture systems have the same impact on bovine embryo transcriptome. Reproduction. 2017;154(5):695–710. doi: 10.1530/REP-17-0449 28982934

[31] Dembélé D, Kastner P. Fold change rank ordering statistics: a new method for detecting differentially expressed genes. BMC Bioinformatics. 2014;15:14. doi: 10.1186/1471-2105-15-14 24423217 PMC3899927

[32] Larionov A, Krause A, Miller W. A standard curve based method for relative real time PCR data processing. BMC Bioinformatics. 2005;6:62. doi: 10.1186/1471-2105-6-62 15780134 PMC1274258

[33] Lesueur J, Walachowski S, Barbey S, Cebron N, Lefebvre R, Launay F. Standardized whole blood assay and bead-based cytokine profiling reveal commonalities and diversity of the response to bacteria and TLR ligands in cattle. Front Immunol. 2022;13:871780. 35677047 10.3389/fimmu.2022.871780 PMC9169910

[34] Diskin MG, Parr MH, Morris DG. Embryo death in cattle: an update. Reprod Fertil Dev. 2011;24(1):244–51. doi: 10.1071/RD11914 22394965

[35] Malhi PS, Adams GP, Singh J. Bovine model for the study of reproductive aging in women: follicular, luteal, and endocrine characteristics. Biol Reprod. 2005;73(1):45–53. doi: 10.1095/biolreprod.104.038745 15744017

[36] Hori K, Matsuyama S, Nakamura S, Iwata H, Kuwayama T, Miyamoto A, et al. Age-related changes in the bovine corpus luteum function and progesterone secretion. Reprod Domest Anim. 2019;54(1):23–30. doi: 10.1111/rda.13303 30085372

[37] Butkiewicz AF, Amaral A, Cerveira-Pinto M, Kordowitzki P. Assessing the influence of maternal age in bovine embryos and oocytes: a model for human reproductive aging. Aging Dis. 2024;16(2):757–68. doi: 10.14336/AD.2024.0305 38916737 PMC11964423

[38] Cree LM, Hammond ER, Shelling AN, Berg MC, Peek JC, Green MP. Maternal age and ovarian stimulation independently affect oocyte mtDNA copy number and cumulus cell gene expression in bovine clones. Hum Reprod. 2015;30(6):1410–20. doi: 10.1093/humrep/dev066 25820694

[39] Alvarez RH, Duarte KMR, Carvalho JBP, Rocha CC, Junior GAA, Trevisol E, et al. Ovarian morphology and follicular dynamics associated with ovarian aging in Bos indicus beef cows. Anim Reprod Sci. 2023;254:107279. doi: 10.1016/j.anireprosci.2023.107279 37353462

[40] Parr MH, Scully S, Lonergan P, Evans ACO, Crowe MA, Diskin MG. Establishment of critical timing of progesterone supplementation on corpus luteum and embryo development in beef heifers. Anim Reprod Sci. 2017;180:1–9. doi: 10.1016/j.anireprosci.2017.02.005 28258785

[41] Devesa-Peiro A, Sebastian-Leon P, Parraga-Leo A, Pellicer A, Diaz-Gimeno P. Breaking the ageing paradigm in endometrium: endometrial gene expression related to cilia and ageing hallmarks in women over 35 years. Human Reproduction. 2022;37(4):762–76. 35085395 10.1093/humrep/deac010

[42] Lucas ES, Vrljicak P, Muter J, Diniz-da-Costa MM, Brighton PJ, Kong C-S, et al. Recurrent pregnancy loss is associated with a pro-senescent decidual response during the peri-implantation window. Commun Biol. 2020;3(1):37. doi: 10.1038/s42003-020-0763-1 31965050 PMC6972755

[43] Salilew-Wondim D, Hölker M, Rings F, Ghanem N, Ulas-Cinar M, Peippo J, et al. Bovine pretransfer endometrium and embryo transcriptome fingerprints as predictors of pregnancy success after embryo transfer. Physiol Genomics. 2010;42(2):201–18. doi: 10.1152/physiolgenomics.00047.2010 20388838

[44] Wang J, Wang S, Chen L, Tan J. SCARA5 suppresses the proliferation and migration, and promotes the apoptosis of human retinoblastoma cells by inhibiting the PI3K/AKT pathway. Mol Med Rep. 2021;23(3):202. doi: 10.3892/mmr.2021.11841 33495818 PMC7821225

[45] Jumai K, Zhang T, Qiao B, Ainiwaer J, Zhang H, Hou Z, et al. Highly Expressing SCARA5 Promotes Proliferation and Migration of Esophageal Squamous Cell Carcinoma. Wang F, editor. J Immunol Res. 2022;2022:1–21. 10.1155/2022/2555647 PMC9232322 35755171

[46] Zou Y, Li J, Su H, Dechsupa N, Liu J, Wang L. Mincle as a potential intervention target for the prevention of inflammation and fibrosis (Review). Mol Med Rep. 2024;29(6):103. doi: 10.3892/mmr.2024.13227 38639174 PMC11058355

[47] Choi Y, Kim H-R, Lim EJ, Park M, Yoon JA, Kim YS, et al. Integrative Analyses of Uterine Transcriptome and MicroRNAome Reveal Compromised LIF-STAT3 signaling and progesterone response in the endometrium of patients with recurrent/repeated implantation failure (RIF). PLoS One. 2016;11(6):e0157696. doi: 10.1371/journal.pone.0157696 27304912 PMC4909214

[48] Suen AA, Jefferson WN, Wood CE, Padilla-Banks E, Bae-Jump VL, Williams CJ. SIX1 Oncoprotein as a Biomarker in a Model of Hormonal Carcinogenesis and in Human Endometrial Cancer. Mol Cancer Res. 2016;14(9):849–58. doi: 10.1158/1541-7786.MCR-16-0084 27259717 PMC5025359

[49] Rafiq A, Aashaq S, Jan I, Beigh MA. SIX1 transcription factor: A review of cellular functions and regulatory dynamics. Int J Biol Macromol. 2021;193(Pt B):1151–64. doi: 10.1016/j.ijbiomac.2021.10.133 34742853

[50] Rutanen EM, Partanen S, Pekonen F. Decidual transformation of human extrauterine mesenchymal cells is associated with the appearance of insulin-like growth factor-binding protein-1. J Clin Endocrinol Metab. 1991;72(1):27–31. doi: 10.1210/jcem-72-1-27 1702447

[51] Cerro JA, Pintar JE. Insulin-like growth factor binding protein gene expression in the pregnant rat uterus and placenta. Dev Biol. 1997;184(2):278–95. doi: 10.1006/dbio.1997.8533 9133435

[52] Altmäe S, Martínez-Conejero JA, Salumets A, Simón C, Horcajadas JA, Stavreus-Evers A. Endometrial gene expression analysis at the time of embryo implantation in women with unexplained infertility. Mol Hum Reprod. 2010;16(3):178–87. doi: 10.1093/molehr/gap102 19933690

[53] Minten MA, Bilby TR, Bruno RGS, Allen CC, Madsen CA, Wang Z. Effects of fertility on gene expression and function of the bovine endometrium. PLoS ONE. 2013;8(8):e69444. doi: 10.1371/journal.pone.0069444 PMC3734181 23940519

[54] Berdiaki A, Zafeiropoulou S, Makrygiannakis F, Drakopoulos P, Gurgan T, Makrigiannakis A. Ageing, a modulator of human endometrial stromal cell proliferation and decidualization: a role for implantation?. Reprod Biomed Online. 2022;45(2):202–10. doi: 10.1016/j.rbmo.2022.03.028 35773140

[55] Derbala Y, Elazzamy H, Bilal M, Reed R, Salazar Garcia MD, Skariah A, et al. Mast cell-induced immunopathology in recurrent pregnancy losses. Am J Reprod Immunol. 2019;82(1):e13128. doi: 10.1111/aji.13128 31006153

[56] Kawamura T, Tomari H, Onoyama I, Araki H, Yasunaga M, Lin C, et al. Identification of genes associated with endometrial cell ageing. Mol Hum Reprod. 2021;27(2):gaaa078. doi: 10.1093/molehr/gaaa078 33258951

[57] Tríbulo P, Siqueira LGB, Oliveira LJ, Scheffler T, Hansen PJ. Identification of potential embryokines in the bovine reproductive tract. J Dairy Sci. 2018;101(1):690–704. doi: 10.3168/jds.2017-13221 29128220

[58] Bae H, Lim W, Bazer FW, Whang K-Y, Song G. Mitigation of ER-stress and inflammation by chemokine (C-C motif) ligand 21 during early pregnancy. Dev Comp Immunol. 2019;94:73–84. doi: 10.1016/j.dci.2019.01.016 30711450

[59] Pereira G, Guo Y, Silva E, Silva MF, Bevilacqua C, Charpigny G, et al. Subclinical endometritis differentially affects the transcriptomic profiles of endometrial glandular, luminal, and stromal cells of postpartum dairy cows. J Dairy Sci. 2022;105(7):6125–43. doi: 10.3168/jds.2022-21811 35636998

[60] Han L, Zhang L. CCL21/CCR7 axis as a therapeutic target for autoimmune diseases. Int Immunopharmacol. 2023;121:110431. doi: 10.1016/j.intimp.2023.110431 37331295

[61] Tanikawa N, Ohtsu A, Kawahara-Miki R, Kimura K, Matsuyama S, Iwata H, et al. Age-associated mRNA expression changes in bovine endometrial cells in vitro. Reprod Biol Endocrinol. 2017;15(1):63. doi: 10.1186/s12958-017-0284-z 28806906 PMC5556672

[62] Borges AM, Healey GD, Sheldon IM. Explants of intact endometrium to model bovine innate immunity and inflammation ex vivo. Am J Reprod Immunol. 2012;67(6):526–39. doi: 10.1111/j.1600-0897.2012.01106.x 22324889

[63] Cronin JG, Turner ML, Goetze L, Bryant CE, Sheldon IM. Toll-like receptor 4 and MYD88-dependent signaling mechanisms of the innate immune system are essential for the response to lipopolysaccharide by epithelial and stromal cells of the bovine endometrium. Biol Reproduction. 2012;86(2). 10.1095/biolreprod.111.092718 PMC4396703 22053092

[64] Kobayashi H, Nishio M, Umetani M, Shigetomi H, Imanaka S, Hashimoto H. Endometrial aging and reproductive decline: the central role of mitochondrial dysfunction. Int J Mol Sci. 2025;26(11):5060. doi: 10.3390/ijms26115060 40507871 PMC12154470

[65] Maekawa R, Taketani T, Mihara Y, Sato S, Okada M, Tamura I, et al. Thin endometrium transcriptome analysis reveals a potential mechanism of implantation failure. Reprod Med Biol. 2017;16(2):206–27. doi: 10.1002/rmb2.12030 29259471 PMC5661823

[66] Forde N, Carter F, Spencer TE, Bazer FW, Sandra O, Mansouri-Attia N. Conceptus-induced changes in the endometrial transcriptome: how soon does the cow know she is pregnant? Biol Reproduction. 2011;85(1):144–56. 10.1095/biolreprod.110.090019 21349821

[67] O S a n d r a, Charpigny G, Galio L, Hue I. Preattachment embryos of domestic animals: insights into development and paracrine secretions. Annu Rev Anim Biosci. 2017;5(1):205–28. 27959670 10.1146/annurev-animal-022516-022900

[68] Chankeaw W, Lignier S, Richard C, Ntallaris T, Raliou M, Guo Y, et al. Analysis of the transcriptome of bovine endometrial cells isolated by laser micro-dissection (1): specific signatures of stromal, glandular and luminal epithelial cells. BMC Genomics. 2021;22(1):451. doi: 10.1186/s12864-021-07712-0 34139994 PMC8212485

[69] Gray CA, Taylor KM, Ramsey WS, Hill JR, Bazer FW, Bartol FF, et al. Endometrial glands are required for preimplantation conceptus elongation and survival. Biol Reprod. 2001;64(6):1608–13. doi: 10.1095/biolreprod64.6.1608 11369585

[70] Matt DW, Sarver PL, Lu JK. Relation of parity and estrous cyclicity to the biology of pregnancy in aging female rats. Biol Reprod. 1987;37(2):421–30. doi: 10.1095/biolreprod37.2.421 3676396

[71] Urzua U, Chacon C, Lizama L, Sarmiento S, Villalobos P, Kroxato B, et al. Parity history determines a systemic inflammatory response to spread of ovarian cancer in naturally aged mice. Aging Dis. 2017;8(5):546–57. doi: 10.14336/AD.2017.0110 28966800 PMC5614320

[72] Ulrich ND, Vargo A, Ma Q, Shen Y chi, Bazzano D, Hannum DF. Proc Natl Acad Sci USA. 2024;121(45):e2404775121. 10.1073/pnas.2404775121 PMC11551439 39471215

