# Supplementary figures and images for: Ageing-driven molecular and functional changes in the bovine endometrium
Source: PLoS One. 2025 Sep 26;20(9):e0332176. doi: 10.1371/journal.pone.0332176 (PMC12468982; doi:10.1371/journal.pone.0332176)

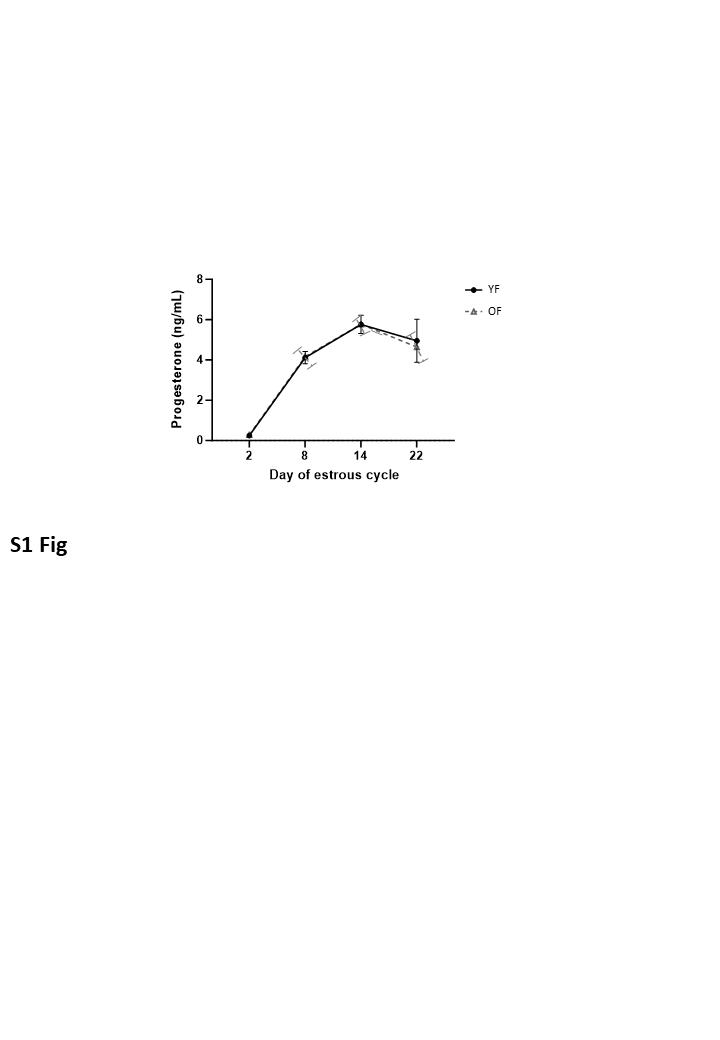

Supplement: S1 Fig — Blood samples were collected on Days 2, 8, 14 and 22 of the estrous cycle on young females (YF, n = 6) and old females (OF, n = 7) included in the transcriptomic analyses and validation by RT-qPCR. Mean + /- SEM. (TIF) [file pone.0332176.s001.tif]

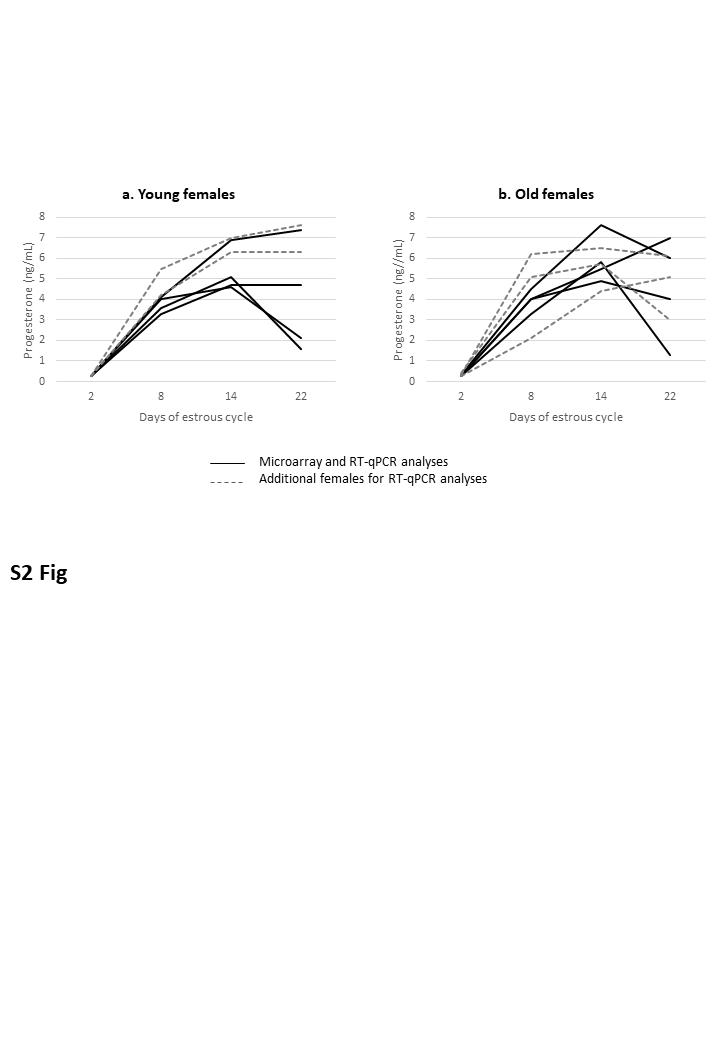

Supplement: S2 Fig — Blood progesteronemia was measured at Days 2, 8, 14, and 22. For each young female (YF); For each old female (OF). Solid lines indicate females included in transcriptomic analyses and RT-qPCR confirmation (n = 4 per group). Dashed lines indicate additional females for RT-qPCR confirmation (n = 2 YF and n = 3 OF). In total, transcriptomic analyses were performed with 4 females per group, RT-qPCR confirmation was performed with 6 YF and 7 OF. (TIF) [file pone.0332176.s002.tif]

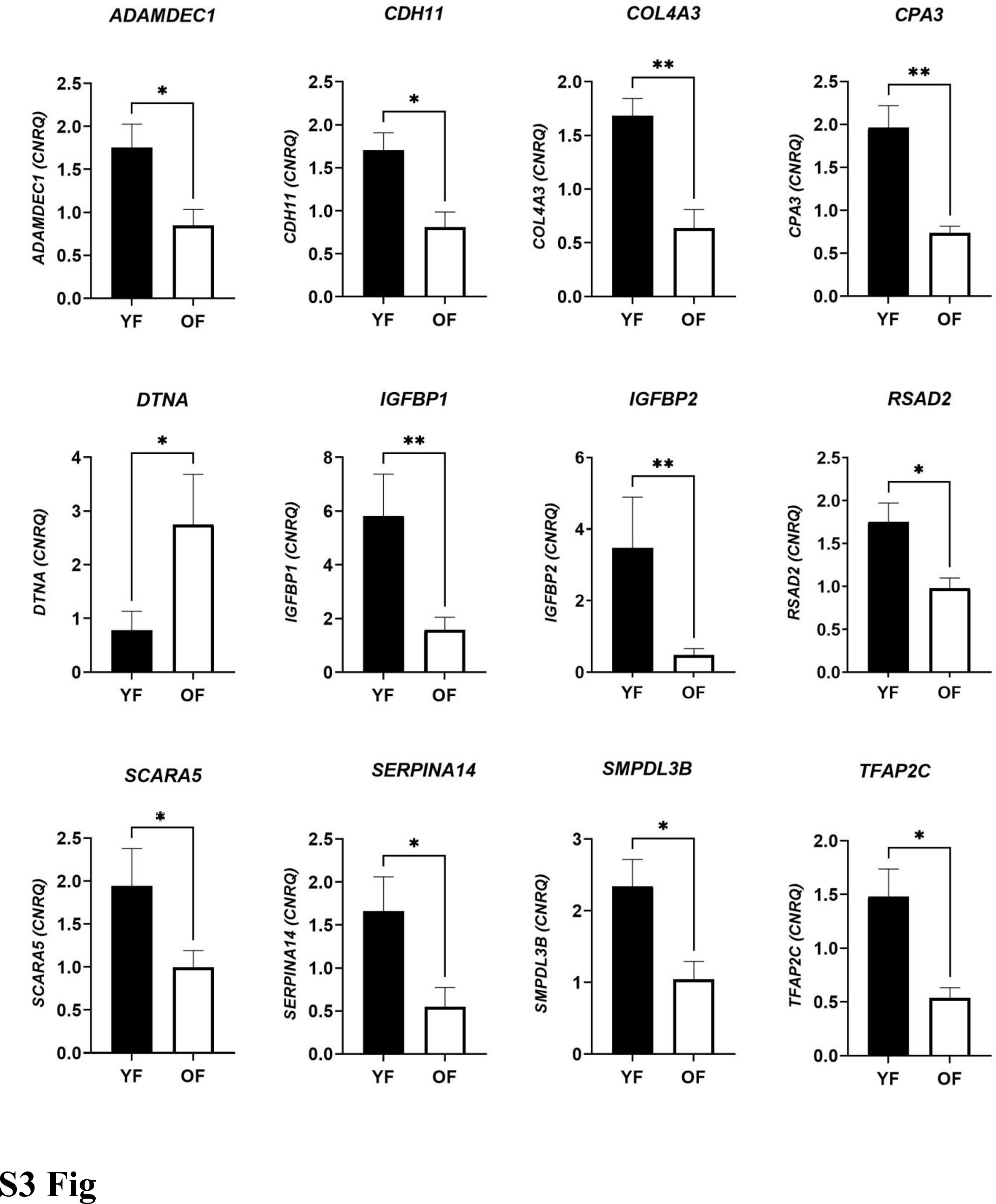

Supplement: S3 Fig — Expression levels of the transcripts were quantified by RT-qPCR using total RNA extracted from endometrial biopsies collected on Day 15 of the estrous cycle from 6 young females (YF, black bars) and 7 old females (OF, open bars). Expression levels for each gene were determined in calibrated normalized relative quantities (CNRQ) and were presented as mean + /- SEM. P value was determined by Mann-Whitney test. * P ≤ 0.05; ** P ≤ 0.01. (TIF) [file pone.0332176.s003.tif]

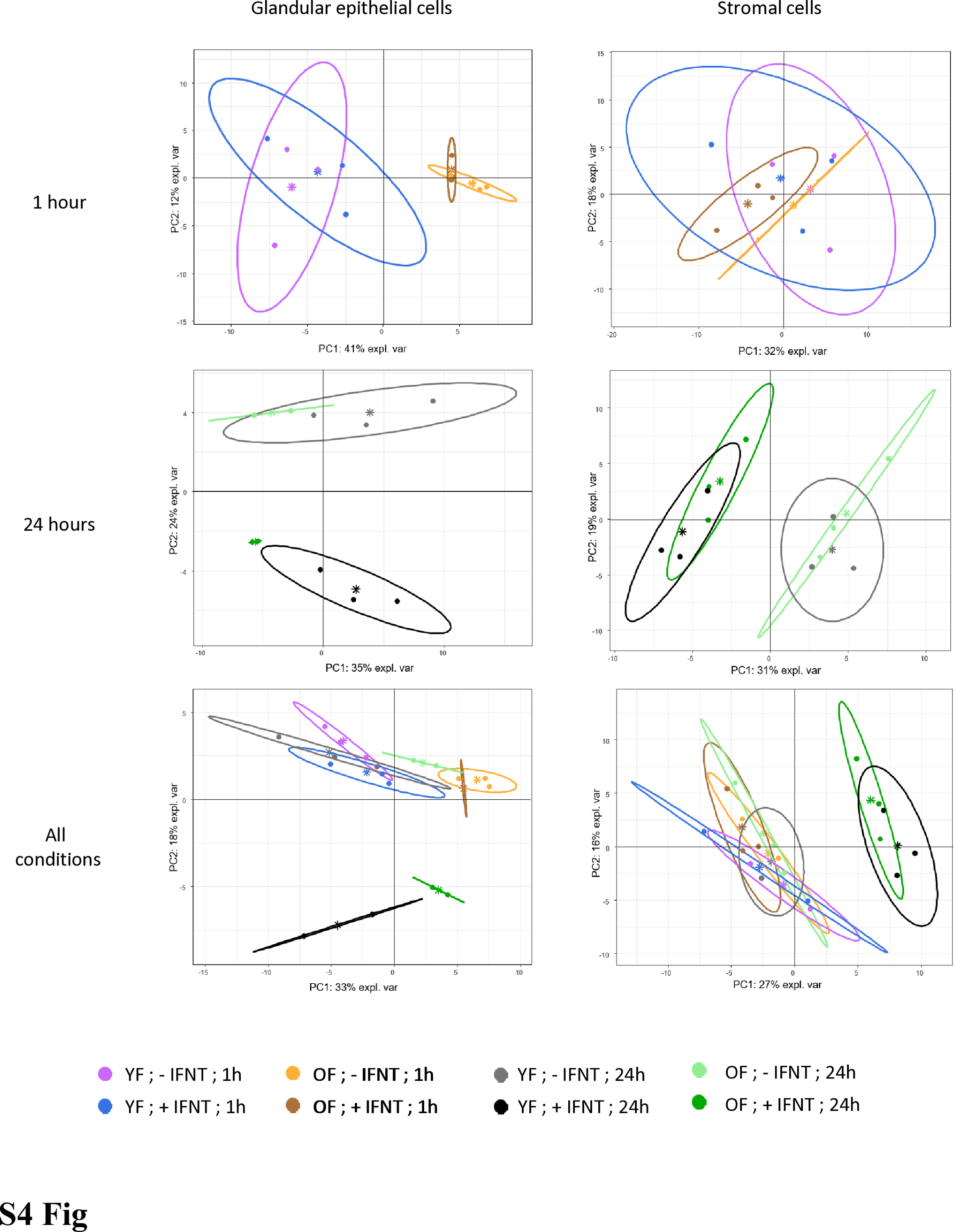

Supplement: S4 Fig — Principal component analyses were performed using the expression levels of 77 and 79 candidate genes quantified in 3 young females (YF) and 3 old females (OF) in glandular epithelial cells and stromal cells, respectively. Each point represents a female. Each asterisk represents the centroid of a group. (TIF) [file pone.0332176.s004.tif]
