# Supplementary material for: Ageing-driven molecular and functional changes in the bovine endometrium
Source: PLoS One. 2025 Sep 26;20(9):e0332176. doi: 10.1371/journal.pone.0332176 (PMC12468982; doi:10.1371/journal.pone.0332176)
Supplement: S1 Table — Exp. 1: experiment 1 (Day 15 of the estrous cycle); Exp. 2: experiment 2 (Day 3 of the estrous cycle). (DOCX) [file pone.0332176.s005.docx]

|  |  | **Animals ID** | | | | | | | | | | | | | | |
| --- | --- | --- | --- | --- | --- | --- | --- | --- | --- | --- | --- | --- | --- | --- | --- | --- |
|  |  | **Young females** | | | | | | | **Old females** | | | | | | | |
|  |  | 1069 | 1070 | 1072 | 1127 | 1101 | 1125 | 1126 | 0233 | 0234 | 0245 | 2258 | 0239 | 0244 | 0247 | 2256 |
|  | Analyses |  |  |  |  |  |  |  |  |  |  |  |  |  |  |  |
| Exp. 1 | Progesterone assay | X | X | X | X | X | X |  | X | X | X | X | X | X | X | X |
|  | Endometrial biopsy: microarray | X | X | X | X |  |  |  | X | X | X | X |  |  |  |  |
|  | Endometrial biopsy: RT-qPCR | X | X | X | X | X | X |  | X | X | X | X | X | X | X |  |
|  | Primary culture of endometrial cells -/+ IFNT ; RT-qPCR |  | X | X | X |  |  |  |  |  | X | X |  |  |  | X |
| Exp. 2 | Progesterone assay | X |  | X | X | X |  | X | X |  | X |  | X |  |  | X |
|  | Culture of endometrial explants -/+ LPS ; Cytokines | X |  | X | X | X |  | X | X |  | X |  | X |  |  | X |
|  | Culture of endometrial explants -/+ LPS ; RT-qPCR | X |  | X | X | X |  | X | X |  | X | X |  |  |  | X |
